# The Great Silk Alternative: Multiple Co-Evolution of Web Loss and Sticky Hairs in Spiders

**DOI:** 10.1371/journal.pone.0062682

**Published:** 2013-05-01

**Authors:** Jonas O. Wolff, Wolfgang Nentwig, Stanislav N. Gorb

**Affiliations:** 1 Functional Morphology and Biomechanics, Zoological Institute, University of Kiel, Kiel, Germany; 2 Institute of Ecology and Evolution, University of Bern, Baltzerstrasse, Bern, Switzerland; Oxford Brookes University, United Kingdom

## Abstract

Spiders are the most important terrestrial predators among arthropods. Their ecological success is reflected by a high biodiversity and the conquest of nearly every terrestrial habitat. Spiders are closely associated with silk, a material, often seen to be responsible for their great ecological success and gaining high attention in life sciences. However, it is often overlooked that more than half of all Recent spider species have abandoned web building or never developed such an adaptation. These species must have found other, more economic solutions for prey capture and retention, compensating the higher energy costs of increased locomotion activity. Here we show that hairy adhesive pads (scopulae) are closely associated with the convergent evolution of a vagrant life style, resulting in highly diversified lineages of at least, equal importance as the derived web building taxa. Previous studies often highlighted the idea that scopulae have the primary function of assisting locomotion, neglecting the fact that only the distal most pads (claw tufts) are suitable for those purposes. The former observations, that scopulae are used in prey capture, are largely overlooked. Our results suggest the scopulae evolved as a substitute for silk in controlling prey and that the claw tufts are, in most cases, a secondary development. Evolutionary trends towards specialized claw tufts and their composition from a low number of enlarged setae to a dense array of slender ones, as well as the secondary loss of those pads are discussed further. Hypotheses about the origin of the adhesive setae and their diversification throughout evolution are provided.

## Introduction

Spiders are, besides insects and mites, the most successful terrestrial arthropods and are of superior ecological importance as predators [1], [2]. Their ecological success is reflected in a high biodiversity [3], [4], the conquest of nearly every terrestrial habitat [5] as well as high densities and biomass production in those habitats [1]. As active predators spiders have the ability to capture and manipulate agile prey, which often are capable of causing severe injuries. Studies on spiders often emphasize web building behaviour, as silk use has reached superior specialization and predatory success in these animals (i. e. [6]). However, silken materials are well-adapted for static adhesion. Once applicated they are restricted to a specific foraging site. Damages in the web (occurring through prey capture or disturbances) have to be repaired with new synthesised silk. Depending on the adhesive properties of the silk a high amount of silken material has to be applied to get an effective trap [7]. This is especially the case in sheet or funnel webs, which are the basic trapping webs. A change of the foraging site is therefore associated with high costs in those spiders [8]. The evolution of viscid silk and orb webs radically reduced the web associated costs and therefore led to an enormous radiation and success of those lineages [9], [10]. However, another strategy was at least equally successful: Many spider lineages never developed a silk-dependent prey capture mode or have independently abandoned web building and explored alternative hunting strategies [9], resulting in more than one half of all Recent spider species hunting without webs [10].

**Figure 1:**
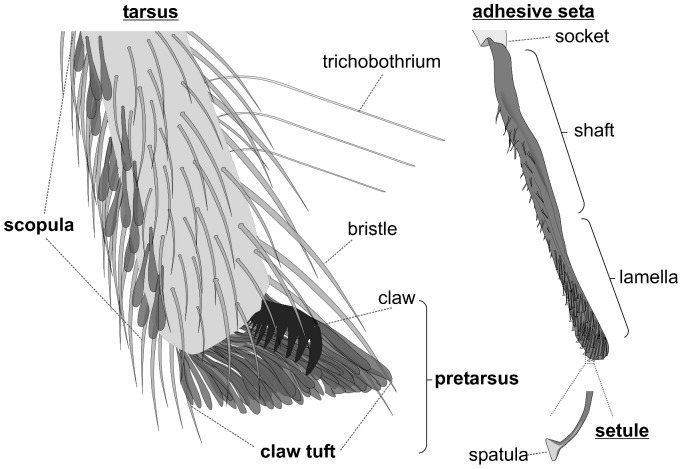
Schematic illustration of a distal spider tarsus bearing scopula and claw tuft. The adhesive setae are coloured in dark grey. In a living specimen they usually appear dark, with the lamellate part being translucent and with an iridescent lustre on the adhesive side.

Free hunting lifestyles implicate alternative mechanisms for capturing, securing and handling of prey. Hairy adhesive pads (scopulae, Fig. 1) located on spider extremities have been previously hypothesized to aid in controlling the struggling prey [11], [12]. High speed video recordings [11], [13], [14] and experimental manipulation [11], [15] have shown the use of adhesive scopulae in prey capture. The lamelliform setae that scopulae are composed of, were shown to produce high adhesion [16] and friction on smooth [17], [18] and rough surfaces [19]. Furthermore, the distal most pretarsal scopulae (claw tufts, Fig. 1) were repeatedly reported to be responsible for impressive climbing abilities 16,17,18,19,20,21,22,23,24, presumably supporting the wandering lifestyle. Thus, we may hypothesize that the evolution of adhesive setae is a valid alternative to the development of snares and is strongly associated with the loss of web based foraging.

Although it was previously mentioned that scopulae and claw tufts primarily are found in free hunting spiders [11], this was neither systematically studied nor set in a phylogenetic context. Furthermore, adhesive setae were found to exhibit a variety of shapes [25], sometimes used in spider classification (i.e. [26]), although it was hypothesized, that different shapes of setae and their organization types evolved convergently due to similar functional demands [11], [23], [27]. However, this was never further studied. Thus, it remains unknown, (1) whether the adhesive setae occurring in spiders are homologous, (2) where they originate from and (3) which pad organisation and setal types are the more basic and which are more derived. This is especially important as spider adhesive pads are central in ecological [28], [29], [30], behavioural [11], [12], [13], [14], [15], [31], taxonomic [2], [23], [26], [27], [32], biomechanical [16], [17], [18], [19], [20], [24], [33], and biomimetical research [34], [35], [36], [37], [38]. We studied the distribution and morphology of adhesive setae phylogenetically to resolve homology, origin and derivation.

For our understanding of the relationship between morphology and function as well as the use of morphological characters in taxonomy, it is important to clearly separate the influence of functional demands and evolutionary constrains. It was previously demonstrated, for the density of the spatulate contact elements of hairy attachment devices in geckoes, insects and arachnids, that this is of high importance for conclusions on functional effects [39]. Therefore we independently compared setal sizes between families, groups of similar body size and groups of similar microhabitat preferences. We hypothesize that differences in setal sizes are a result of phylogeny, not scaling effects or ecological adaptation as previously stated [25], [40].

Due to the recent progress in resolving the phylogeny of spiders and the developments in bioinformatics and statistics, evolutionary paths of single characters can be reconstructed, getting closer to resolving the evolution of adhesive pads in spiders. Here we tested the previous hypothesis that adhesive setal pads are restricted to free hunting spiders [5], [15]. Evolutionary trends in pad organisation and setal shapes, as well as the origin of adhesive setae are discussed. Furthermore, we present the first comprehensive study of the distribution of adhesive setae among the Araneae, indicating their evolutionary success and high ecological importance.

## Methods

### Morphological Studies

Spider legs (anterior and posterior) were studied by means of stereo microscopy, scanning electron microscopy (SEM) on air dried, critical point dried and cryo dried samples, and transmission electron microscopy (TEM). Additionally, morphological data was accessed from the *Spider AToL* project, provided on morphbank :: biological imaging (Florida State University, Department of Scientific Computing, Tallahassee, USA; http://www.morphbank.net) under cc 3.0 (by-nc-sa) license. Further, primary literature data were included. A list of all analyzed spider species and corresponding references can be found in the supplementary material (Figure S1).

**Figure 2:**
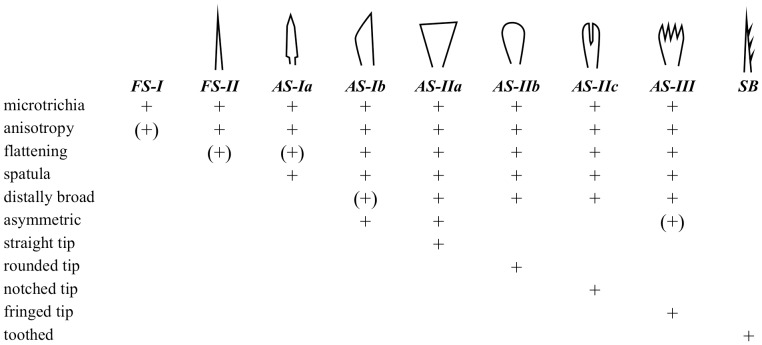
Characters of setal types occurring in the distal tarsus/claw tuft. For detailed description see [25]. AS, adhesive seta (with spatulae); FS, frictional seta (without spatulae); SB, serrated bristle (see [55]).

The presence and the types of adhesive setae and their organisational variety (scopula, claw tuft) were documented. Adhesive setae (AS) are defined as setae featuring spatulate microtrichia (see [25] for details). Scopula (sc) is defined as a group of adhesive setae at the leg, regardless of setal density. Claw tuft (ct) is defined as a dense array of adhesive setae located at the distal tarsal tip, being clearly delimited from the scopula and situated on a rather flat, articulated plate (‘tenent plate’ [25], [31], [32]) or, at least, emerging from the pretarsal membranous area (onychium). Clearly delimited scopulate parts, located at the distal ventral tarsus but not emerging from the pretarsal parts, are termed ‘false’ claw tufts (f-ct). Setal types are classified according to the shape of the distal lamella (Fig. 2; see [25] for details). For part of the material studied a closer inspection of the fine structure was done, including information on (1) the spatula shape, (2) the structure of setal backing, and (3) the cross section of the setal lamella. Based on the SEM images, morphometric measurements (seta width, spatula width) were performed on the adhesive setae of claw tufts and ‘false’ claw tufts.

### Meta Analysis

Species numbers and taxonomic information were taken from Platnick [3]. Phylogeny is based on Coddington [2], [9], including key findings of further new studies (for details, see [30]).

For each analyzed species foraging guild was defined as follows: (wb) Spiders that build webs, with the function to capture and immobilize the prey due to adhesion (viscid or cribellar silk in orb webs and cob webs) or entangling (sheet webs and funnel webs). That means the web causes a reduction of mobility of the prey, which facilitates getting overwhelmed by the spider. Some spiders were included here, which capture the prey with silks by bonding it while running around (Oecobiidae, Hersiliidae) or spitting glue onto the prey (Scytodidae). (fh) Spiders that do not build prey capture webs, but primarily grasp and capture the prey directly with the legs. This includes spiders that only use silk threads or webs for prey sensing (i. e. trapdoor spiders), shelter or reproduction and those that do not build any web. Data on foraging behaviour was obtained from Joqué and Dippenaar-Schoeman [4], Cardoso et al. [41] and the primary literature (Figure S1).

For each spider family included, the proportion of each pad type (scopulae, claw tufts, both, none) and the foraging guild (wb, fh) among the investigated species was calculated and both separately multiplied with the total species number. Meta data and the corresponding references are included in the species list provided in the supplementary material (Figure S1).

To find indications for parallel evolutionary processes Ancestral State Reconstruction was performed with *Mesquite* [42] using the parsimony principle. For this purpose traced characters were binary coded: (1) spatulae ( = adhesive setae in general); (2) scopulae; (3) ‘false’ claw tufts; (4) claw tufts; (5) web abandoning; (6) the occurrence of setal type dominant in the distal tarsus (each setal type analyzed separately).

Morphometric data on (‘false’) claw tuft setae were set in context with the body size, microhabitat preferences and phylogeny (see below) to separate influences of scaling effects, ecological adaptation and phylogenetic restrictions. Body size was either measured in the samples (body length from the chelicerae to the spinnerets) or such information was taken from literature. Ecological data were obtained from Joqué and Dippenaar-Schoeman [4], Cardoso et al. [41], Nentwig et al. [43] and the primary literature. For statistical analysis R software package [44] was used.

## Results

### Phylogenetic Analysis

Adhesive setae (spatulae) evolved at least eight times in spiders, resulting in the clustered occurrence of the trait. (1) Adhesive setae are present in the derived Mygalomorphae, including Nemesiidae, Cyrtaucheniidae, Paratropidae, Barychelidae, Theraphosidae, and Idiopidae. (2) Among the haplogyne spiders in the superfamily Dysderoidea. (3) Within the superfamily Palpimanoidea the Palpimanidae, Stenochilidae, Huttoniidae, and Archaeidae. (4) Among all dionychian families and the Miturgidae, Ctenidae, Zoropsidae, Psechridae, and Lycosidae within the grade-shaped tapetum clade. (5) Some species of Desidae. (6) Single species of Dictynidae. (8) Species of Tengellidae and Homalonychidae, at the base of the RTA-clade.

**Figure 3:**
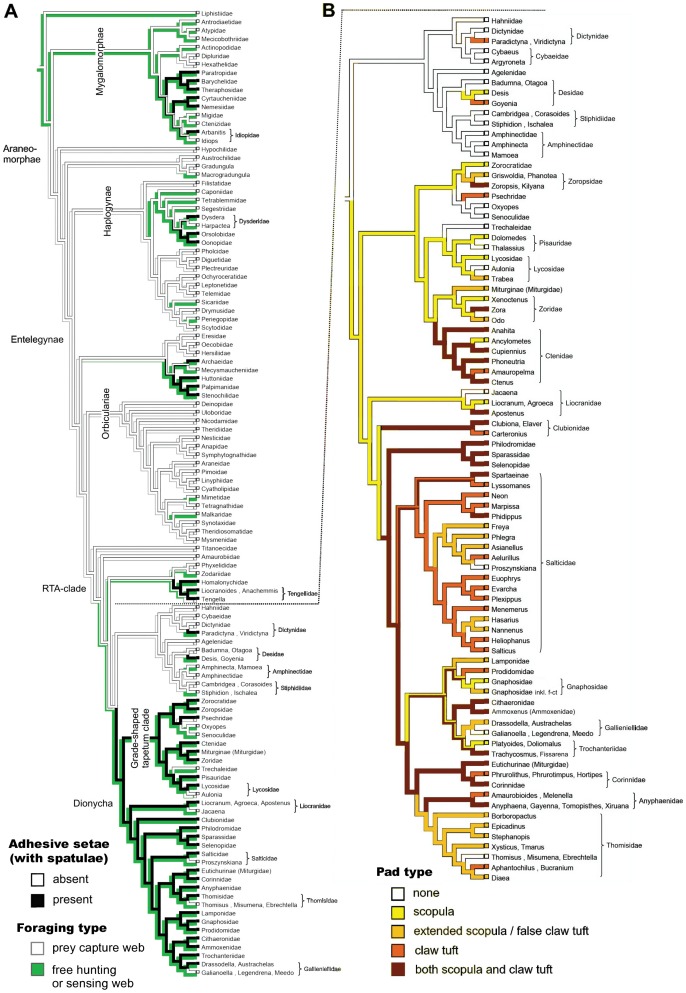
Evolution of hairy adhesive pads and web loss in spiders. A. Phylogenetic relationships among the Araneae, adapted from the most recent literature survey (see [30] for details), and the distribution of adhesive (spatulae-bearing) setal pads and web abandoning. Character traces follow the Ancestral State Reconstruction performed with the Mesquite software. B. Combined character traces of pad type distribution in the RTA-clade. The model clearly suggests an early origin of scopulae and the derived state of claw tufts.

Multiple evolution is supported by the analysis of character traces of spatulae (Fig. 3A), pad types (Fig. 3B), and setal types (Figure S2.A). It is reflected by observed differences in the fine structure of adhesive setae (i. e. shape of the distal lamella, structure of setal backing and shape of the spatula; Figure S3).

**Figure 4:**
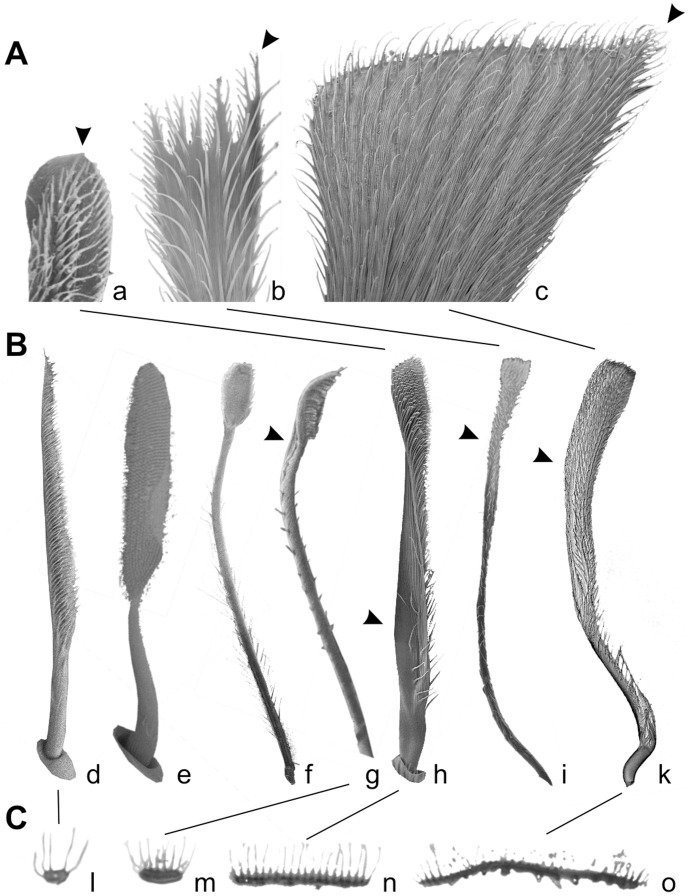
Electron microscopy of isolated setae, different scales. A. SEM micrographs of distal tips of claw tuft setae, rear view. Arrowheads indicate the remaining tapered tip of the expanded setae. a. Adhesive seta type IIb in *Micaria formicaria* (Gnaphosidae). b. Adhesive seta type III in *Clubiona pallidula* (Clubionidae). c. Large adhesive seta type IIa in *Anyphaena accentuata* (Anyphaenidae). B. SEM micrographs of setae, lateral view. Arrowheads indicate the twisted lamella shaft occurring in claw tuft setae. d. Frictional seta type II in *Xysticus lanio* (Thomisidae) ventral tarsus. e. Scopula seta of type IIb in *Clubiona lutescens* (Clubionidae) prolateral tarsus. f. Scopula seta of type IIb in *Palpimanus gibbulus* (Palpimanidae) prolateral metatarsus. g. Brush like claw tuft seta of type Ia in *Homalonychus selenopoides* (Homalonychidae), a presumably primitive character. h. Claw tuft seta of type IIb in *Euophrys frontalis* (Salticidae). i. Claw tuft seta of type III in *Clubiona pallidula*. k. Claw tuft seta of type IIa in *Anyphaena accentuata*. C. TEM micrographs of sections of the distal part of tarsal setae. l. Frictional seta type II in *Nops largus* (Caponiidae). m. Adhesive seta type Ia in *Xysticus cristatus* (Thomisidae). n. Adhesive seta type IIb in *Evarcha arcuata* (Salticidae). o. Adhesive seta type IIa in *Anyphaena accentuata*.

**Figure 5:**
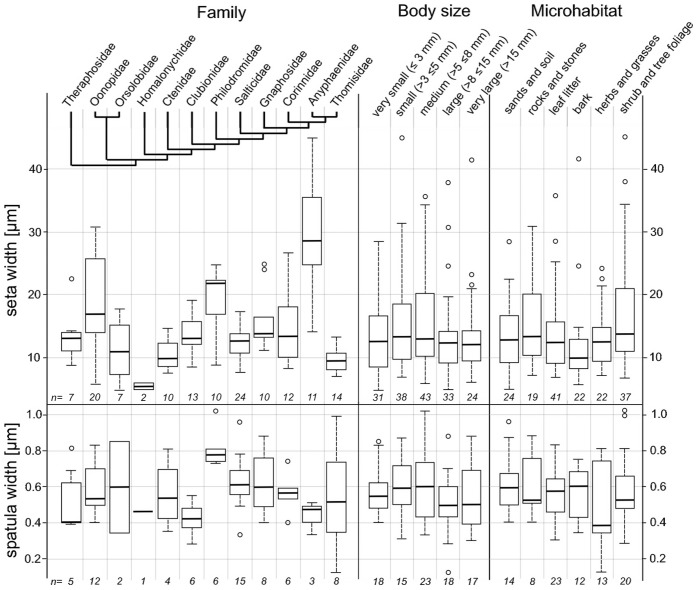
Body size and preferred microhabitat. Box plots showing the 25th and 75th percentiles and the median line; error bars define the 1.5 times interquartile range; rest values are marked by single circles. Numbers at the bottom give the species numbers sampled (each including the mean width of ten randomly chosen setae/spatulae of the distal part of the claw tuft). Seta width differs significantly between species of different families (Kruskal-Wallis rank sum test: p = 0.000), but not between differently sized (p = 0.155) and ecological groups (p = 0.102) of the overall sample. The same holds for spatula size (families: p = 0.030; size = 0.377; microhabitat = 0.860).

Analysis of combined character traces of pad types suggests that scopulae evolved first, followed by claw tuft development. Scopulae are homologous in the derived taxa of the RTA-clade, but claw tufts are not. Similarities in the fine structure of the adhesive setae (esp. setal shape) and the occurrence of intermediate forms (distally extended scopula) indicate that claw tufts previously evolved from scopulae in the mygalomorph lineage, Tengellidae, Desidae, Zoropsidae, Ctenidae, and higher Dionycha, excluding Liocranidae, Clubionidae, and Anyphaenidae. In the latter ones and Zoridae there is a shape of the claw tuft setae divergent from the scopula setae (setae with lateral asymmetry tapered in one direction; Fig. 4A b–c) and a considerably smaller spatula size than in previously mentioned lineages (Fig. 5).

We found cases where scopulae or claw tufts are reduced or lost. Adhesive setae were totally lost in Oxyopidae, Senoculidae, and Trechaleidae, within Pisauridae (*Thalassius*), Liocranidae (*Jacaena*), Salticidae (*Proszynskiana*), Gallieniellidae (*Galianoella*, *Legendrena*, *Meedo*), and Thomisidae (*Thomisus*, *Misumena*, *Ebrechtella*). Scopulae were lost among most salticids (excluding the Spartaeinae and *Phidippus*) and within Ctenidae (*Amauropelma*), Clubionidae (*Carteronius*), Corinnidae (*Phrurolithus*, *Phrurotimpus*, *Hortipes*), and Anyphaenidae (*Amaurobioides*, *Melenella*). Claw tufts are reduced in some salticids (*Freya*, *Asianellus*, *Nannenus*) and totally lost in *Ancylometes* (Ctenidae). For Thomisidae, Anyphaenidae, and the super family Gnaphosoidea, an ancestral loss of claw tufts is assumed, followed by partial regain of this trait.

Flattened tapered setae with an anisotropic coverage of microtrichia, lacking spatulae (FS-II) are widespread in the ventral tarsus of araneomorph spiders. The evolution of this character was reconstructed to be an early event, just before the split into haplogynes and entelegynes (Figure S2.A), thus all setae of this type being homologous among the Araneomorphae. We found setae with a similar structure, but bearing spatulae and thus being adhesive, in *Dysdera* (Dysderidae) and some thomisids, and in the proximal part of claw tufts in zorids and anyphaenids. Thus, we interpret those as directly originating from the FS-II setae.

There is a significant difference of the setal width (Kruskal-Wallis rank sum test: p = 0.000) and spatula width (p = 0.030) between members of different families, which is not reflected by either body size or preferred microhabitat (Fig. 5).

### Distribution of Adhesive Setae

**Figure 6:**
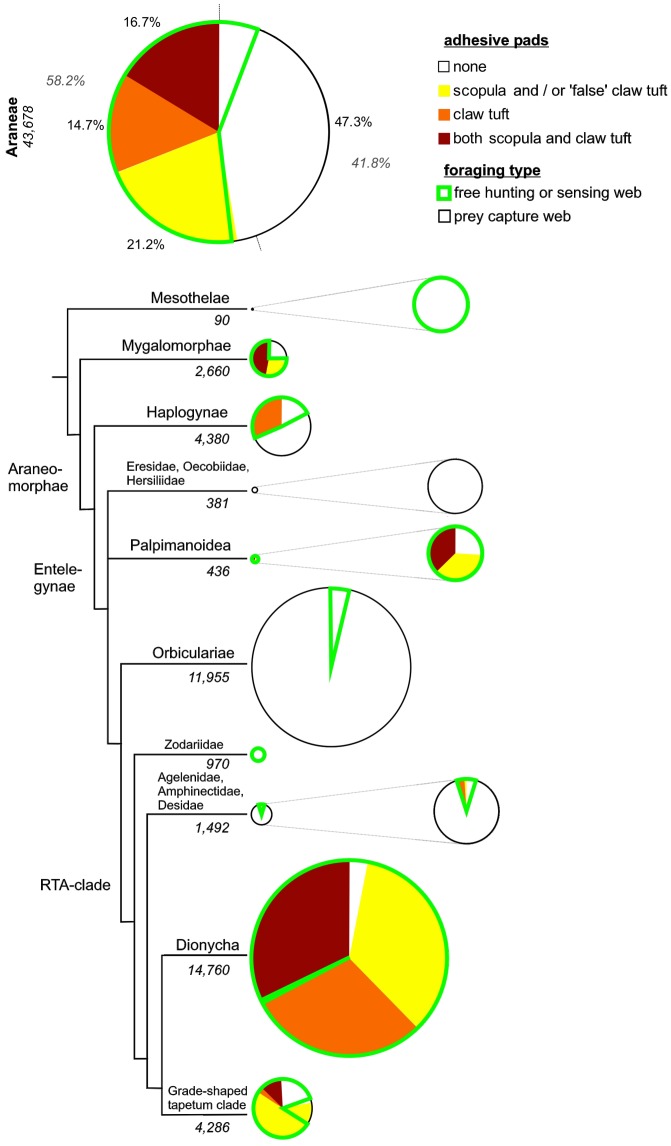
Distribution of adhesive setae in spiders. Above: proportions of species bearing claw tufts, scopulae (incl. ‘false’ claw tufts), both or none, combined with lifestyle. Tree and plots below indicate proportions among the different lineages, with sizes of first plots resembling the number of species (numbers given in italic font). Results show the two major evolutionary pathways of spiders, web builders and free hunters, out of which the latter ones are often associated with adhesive setae.

Slightly more than one half of all spider species are estimated to bear adhesive setae, with 21.2% having scopulae (or/and ‘false’ claw tufts), 14.7% only claw tufts and 16.7% both scopulae and claw tufts (Fig. 6).

Among the Mygalomorphae, adhesive setae are estimated to occur in 72.1% of species, in the Haplogynae 33.4% (84.8% of the Dysderoidea), in the Palpimanoidea 47.4%, the Dionycha 96.6%, the Lycosoidea 78.1% and the Agelenoidea 6.6%.

According to the lifestyle 82.8% of free hunting spiders feature adhesive setae, in contrast to only 1.1% of web building spiders. Scopulae and claw tufts each occur in more than half of the free hunting spiders. Among the web building spiders bearing adhesive setae, 75% bear scopulae and 25% claw tufts.

## Discussion

### Exploring Alternative Adhesives

An important demand on a predatory lifestyle is the capability of capturing and securely handling the struggling prey. For this purpose, spiders use silks of high adhesive strength [45], [46] for capturing and immobilising prey. However, this always comes with material and energetic costs [7], [8]. Thus, there must have been a great selective advantage towards the evolution of reusable adhesives.

This hypothesis is well supported by the widespread occurrence of adhesive setae among the phylogenetic tree of spiders and by their convergent evolution within different spider lineages. Evidence for convergent evolution of similar structured adhesive pads was recently presented for insects [47] and gekkotan lizards [48]. Especially the latter are generally analogous in function and structure to spider adhesive pads. However, in geckos these exclusively serve attachment to the substrate and locomotion. Our results on spiders indicate that, with some exceptions, adhesive setae have rarely evolved first in the distal most tarsal parts that make contact with the substrate while walking. The more ancient organization types, such as leg scopulae, are likely to play a major role in prey capture, supporting our hypothesis that the adhesive setae evolved as a substitute for silk capture threads.

There are several arguments that speak for the assumption that scopulae in spiders are generally an adaptation for prey capture. (1) Scopulae are often restricted to or more developed in the anterior legs [11], [25], [28]. (2) Scopulate setae are mainly distributed in the pro- and retro-lateral parts of the tarsus, metatarsus and tibia, whereas at the ventral side they are lacking [11], [23], [25], [28]. (3) Most spiders walk on their pretarsal tips. Thus, scopulate tarsal and metatarsal parts are rarely in contact with the ground substrate (J. Wolff, pers. obs.). (4) The adhesive sides of the scopula setae are often facing away from the ground in resting animals and become erect by increased hemolymph pressure [11], [12] (J. Wolff, pers. obs.). Presumably, it leads to the activation of the adhesive function of the setae, however, this statement needs further experimental proof.

In salticids, leg scopulae are only reported from the araneophagous genera *Portia*, *Brettus,* and *Cyrba* [12]. In Palpimanidae they are obviously used to overwhelm the dangerous arachnid prey [14]. Thus, the presence of leg scopulae may facilitate handling oversized and dangerous prey and therefore access new food sources. Other adaptations include the erectable spines (widespread), elongated chelicerae (especially in Archaeidae), and legs (i. e. Sparassidae [11]). These features prevent the bitten prey from getting too close to injure the spider, but being kept from escaping as long as it is still active.

However, scopulae may play a further role in attachment on highly structured or uneven substrates (such as coniferous foliage or twigs; J. Wolff, pers. obs.), or in attachment to a mate during copulation, especially in the species with strong sexual dimorphism of scopula distribution, such as some ctenizids [49], philodromids, lycosids (J. Wunderlich, pers. comm.), and even burrowing in sand dwelling species [50].

### From Scopulae to Claw Tufts – Extending the Functional Role of Adhesive Pads

Most previous studies emphasized the locomotory function of the adhesive setae, indicated by the impressive climbing ability of these spiders [16], [17], [19], [20], [21], [22], [23], [24], [33], although this predominantly includes the distal most claw tufts. Rovner proposed that prey capture was the original evolutionary driving force in the evolution of attachment organs in spiders, and that their use in locomotion was a secondary benefit that led to the occupation of new habitats [11]. This idea was also supported by Miller et al. [28].

**Figure 7:**
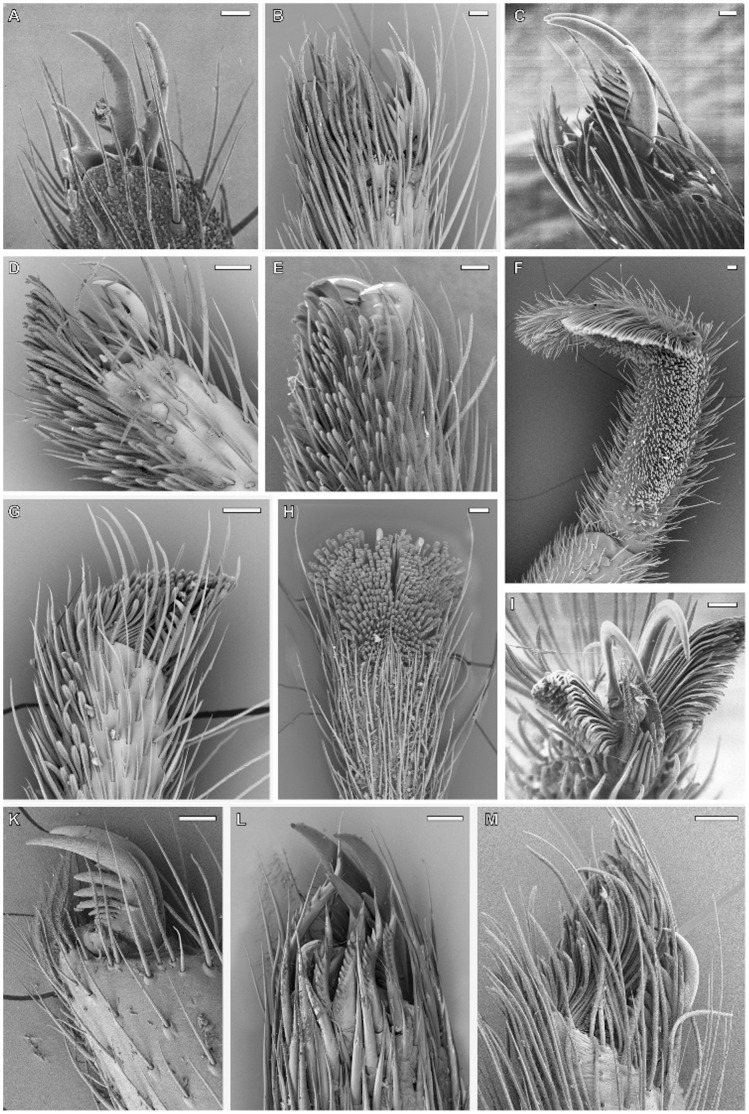
SEM micrographs of the distal portion of spider tarsi bearing the tarsal claws and setal pads; ventral or prolateral view; scale bar - 50 µm. A. Primitive pretarsus in the ancient *Heptathela* sp. (Liphistiidae), juvenile, lacking specialized setae. B. Distal tarsus of *Malthonica ferruginea* (Agelenidae) with a dense ventral coverage of FS-II setae typically occurring in spiders of the basic web types. C. Distal tarsus of the desert dwelling *Sicarius* sp. (Sicariidae), with reduced setal structures. D. Distal tarsus of *Trabea paradoxa* (Lycosidae), showing distally extended scopula, resulting in a primitive foot pad (‘false’ claw tuft). E. Distal tarsus of *Drassodes lapidosus* (Gnaphosidae), with an extended scopula, including a ‘false’ claw tuft. Note the seta width increasing in the distal part of the pad. F. Derived prey capture leg in *Palpimanus gibbulus* (Palpimanidae), bearing the scopula with spatulate setae. G. Distal tarsus of *Clubiona terrestris* (Clubionidae) featuring both scopulae and claw tufts. Note the foot pad emerging from the pretarsus, thus being retracted together with the claws. H. Distal tarsus of *Marpissa muscosa* (Salticidae), bearing only the restricted claw tufts. I. Distal tarsus of the sand-dwelling desert spider *Homalonychus selenopoides* (Homalonychidae), bearing claw tufts with brush-like, non-widened adhesive setae. K. Distal tarsus of *Misumena vatia* (Thomisidae), lacking adhesive setae, a presumed secondary loss. L. Distal tarsus of *Araneus quadratus* (Araneidae), bearing the serrated bristles and the enlarged third claw, adaptations of the derived web building taxa. M. Distal tarsus of the web building *Fecenia cylindrata* (Psechridae), including claw tufts, a great exception in silk trappers.

Untypically for representatives of lycosids, *Trabea paradoxa* is found exclusively on grasses (Grabolle, pers. comm.). In its scopula, a shift of adhesive setae towards the distal part of the tarsus is observed, resulting in the formation of a pad-like structure with a high setal density (Fig. 7D) and in the ability to climb steep glass surfaces (J. Wolff, pers. obs.). Similar observation has been reported for *Rabidosa hentzi*, whose foot pad structures were described as ‘claw tufts’ [28]. However, close inspection of *T. paradoxa* showed the pads emerging from the distal tarsal margin, not the pretarsal region, and the lack of basal plane plates (tenent plates) that define the derived claw tufts [31], [32]. This is assumed to also be the case in *R. hentzi*. The same is true for Gnaphosidae which lack claw tufts, but contain such genera as *Drassodes* and *Micaria* being able to climb on glass due to the presence of a distal extension of the scopula (‘false’ claw tuft; Fig. 7E). This indicates that claw tufts previously evolved from the scopulae among representatives of those lineages. This hypothesis is further supported by a similar structure of scopula and claw tuft setae (both of Type IIb with the rounded tip).

However, at least in zorids, anyphaenids, and clubionids, claw tuft setae could have evolved independently from scopulae, indicated by the differences in their shapes (AS-Ib AS-IIa and AS-III with lateral asymmetry, thus, being still tapered in one direction). Those setae may have directly evolved from the FS-II setae and expanded through an asymmetric broadening of the distal part, which can be reconstructed by the intermediate forms often occurring in the proximal part of those claw tufts [25]. Additionally, the small spatula size recorded supports this hypothesis, as the evolution from small to large spatulae is presumed to be an overall trend, with the exception of strictly ambushing hunters specialized for a particular microhabitat (esp. Thomisidae, see below). In Palpimanidae, the morphology of claw tuft setae differs greatly between and even within genera (*Palpimanus*). Thus, we assume that evolution of this character is highly dynamic within this family, presumably due to the high specialisation of the scopula, generally restricted to the anterior legs (Fig. 7F). The origin of claw tufts is unclear in this case. The same holds for the Dysderoidea, as no scopulae have been recorded in this group.

**Figure 8:**
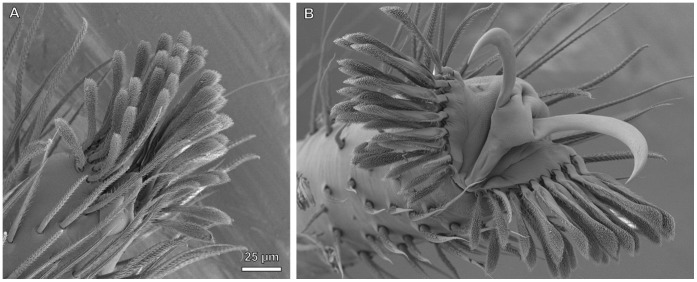
Cryo-SEM micrograph of male *Euophrys frontalis* (Salticidae) pretarsus, showing mechanics of claw tuft spreading. A. Pretarsus with the claw tuft retracted (low hemolymph pressure). B. Claw tuft protracted under high hemolymph pressure, caused by tight squeezing of the femur. Deformation causes a spreading of the divided claw tuft und protraction of the claws, probably important for fast detachment.

The evolution of claw tufts is associated with the formation of a highly sclerotized basal plate (tenent plate [31]) in the pretarsal region, thus being articulated with the tarsus. The latter occurs in the derived lineages of the Dionycha (except gnaphosids, most thomisids and some salticids), Dysderoidea, Theraphosidae, and Ctenidae. It permits spreading and mobility of the pads [24], [31], [51] (Fig. 8). This may facilitate control of both attachment and detachment. The movement of the pretarsus during locomotion has been previously observed in salticids [31].

There are some arguments that speak for a primary locomotory function of the claw tufts differing from the function of the scopulae. (1) Claw tufts typically contact the substrate in walking and climbing spiders. (2) In contrast to the scopulae, claw tufts are always well developed in all the legs of those species which feature them. (3) Whereas scopulae are more developed in the anterior legs, claw tufts are often larger in the posterior legs presumably because these produce the highest forward thrust [25]. (4) Among free hunting spiders those having claw tufts make up a significantly higher proportion found in above-ground and highly structured habitats such as broad leaf litter, than found at ground level and on even substrates (J. Wolff, unpublished). This argument speaks for claw tufts being an adaptation to enhance climbing ability.

For free hunting spiders claw tuft evolution might have been crucial for the colonization of herbs, shrubs and trees. Thus this can be regarded as a key innovation resulting in great radiations of the Dionycha in the Eocene and of the Theraphosidae in the Miocene [52].

Additionally, claw tufts are used for prey capturing, mating, and grooming [31].

For Desidae, Forster proposed an evolutionary path from an initial, single large adhesive seta towards a typical claw tuft with numerous setae of smaller size and the reduction of the third claw [53]. However, it remains unclear, from where those large setae are derived. The idea by Ubick and Vetter of the seta being a derivate of the third claw [27] seems to be erroneous, as this claw is still present in those groups. We assume that it is an enlarged scopula seta, because of its typical setal shape (AS-IIb). This is likely as scopulae are still found in some representatives of the Desidae [53]. The desid genera carrying the enlarged adhesive setae have abandoned web building. The same is the case for two genera of Dictynidae [53]. Both families thus might include species reflecting several states of similar evolutionary processes and are therefore worthy of being studied in more detail.

Indeed the trend from some large setae to numerous slender ones seems to be ubiquitous and we assume the large setae (i.e. in Philodromidae, Anyphaenidae, Ammoxenidae, some Gnaphosidae and Liocranidae and Oonopidae; Fig. 5) to be an ancient trait. In the ‘false’ claw tufts of *Drassodes* and *Micaria* seta width doubles from proximal to distal (Fig. 7E). This can be explained by the restricted space at the distal margin of the tarsus contacting the ground. Thus the beneficial increase of the contact area cannot be achieved solely by an increase of setal number. The formation of the planar tenent plate permitting a very dense array of setal sockets comes with a higher degree of sclerotization to retain stability. Because it emerges from a membranous area, normally lacking any setal structures, this is a more complex transformation and must be interpreted as a derived character. Morphometric comparisons showed that the setal size is primarily an effect of phylogenetic relationships rather than body size or the preferred microhabitat (Fig. 5). Thus, the previous general hypothesis that setal enlargement is an adaptation to smooth substrates [25] should have limitations at a level higher than the family. However, the results of the present paper do not exclude both scaling and adaptation effects within families.

The main functional difference between scopula and claw tuft setae is given by the different target surfaces. If the scopula evolved for prey retention, the targets are small bodies with regularly structured surfaces often including setae (arthropod cuticle). In contrast, claw tufts primarily adhere to plane substrates often including fractal surfaces (surfaces with many superimposed wavelengths, i.e. barks, rocks or some complex specialised plant surfaces). So in claw tuft setae the size of the distal most lamellate part is crucial for establishing contact, whereas scopula setae are assumed to adhere with a great portion of the spatulate part. Furthermore, claw tufts have to resist much more attachment-detachment cycles and provide a stable contact in complex movements (i. e. a sideward turning of the body). Those functional demands resulted in the claw tuft setae being distally broadened and including a twisted lamellate shaft (Fig. 4B g–k). These trends can be observed in most lineages featuring claw tufts.

### Adhesive Setae in Web Building Spiders

Adhesive setae appear almost exclusively in wandering spiders. This indicates that these are adaptations to a free hunting lifestyle. The other major foraging guild, mainly comprising the orb web weavers (Orbiculariae), attained their ecological success through the improvement and diversification of silken materials and web trap structure, achieving higher economical use of silk material [10], [54]. Especially for Orbiculariae the development of scopulae conflicts with higher adaptations to web hunting. For example, their serrated bristles (Fig. 7L) are important for thread grasping [55] and simultaneously prevent sticking to their own web [56]. However, scopulae do not generally disable moving among webs, as they occur in some web building lineages (Psechridae (Fig. 7M), some Lycosidae). Furthermore, movement in webs, even on sticky threads, has been observed to be unproblematic for web invading salticids with well-developed claw tufts [57]. The claw tuft bearing clubionids and salticids are also known for their habit of building silk retreats, in which they can move with ease.

In the case of Psechridae having a claw tuft might indicate that these spiders evolved from a free hunting ancestor and regained web building behaviour later on. This is also indicated by the pseudo-orb web shape [58].

Adhesive setae may even have been evolved from an anti-adhesive device among web building spiders. This is indicated by the widespread occurrence of the spatula-less flattened branched setae (formerly termed frictional setae Type-II (FS-II) [25]) among sheet web weavers (Figure S1), which facilitate movement on the silk sheets [5]. These share some of the derived characters with scopula setae (high directionality of microtrichious coverage, beginning of both broadening and flattening in their distal regions; Fig. 2; Fig. 4B d, C l). Thus, we assume these to be ancestral states for further development of adhesive setae.

### The Secondary Loss of Adhesive Setae

Despite the great advantages in prey capture and climbing, the hairy adhesive pads may also be associated with costs: (1) Attachment and detachment relies on the applied shear forces elicited by muscular activity [18]. Thus, locomotion using claw tufts might rely on higher energy consumption thus limiting maximal running speed. (2) Spatulae are exposed to high mechanical stress and might be damaged due to abrasion wear [19], which then leads to a loss of their efficiency. (3) Adhesive setae might be less effective or even disadvantageous on some substrates, such as plant surfaces with a crystalline wax coverage of small scale roughness [19]. (4) Adhesive setae are very complex structures, which might be associated with higher developmental costs. (5) Moulting problems might occur more frequently, as the scopulae or claw tufts could be entangled within the exuvia. (6) Claw tufts may reduce the efficiency of the claws, because of the basal plates, limiting the freedom of claw movements within the articulation, and the setal array might hinder substrate interlocking by claws. This would cause a loss of attachment ability on highly corrugated surfaces, such as wooden or rocky substrates. This would explain why some groups (i. e. among salticids) exhibit reverse evolution back to ‘false’ claw tufts.

Thus, it is likely that there are trade-offs that should lead to a loss of adhesive setae, when the benefits do not compensate the costs. According to the major functional role, the distribution of scopulae may reflect prey preferences, whereas the occurrence of claw tufts should reflect the habitat preference (climbing demands) and activity of the spider (runner vs. ambusher). Many ground living species, especially in the families of Liocranidae, Ctenidae, Lycosidae, and Gnaphosidae lack claw tufts. On the other hand, they are frequently abundant in the ground-dwelling species of Corinnidae, Clubionidae, Salticidae, Philodromidae, Sparassidae, and even in the burrowing Ammoxenidae, Homalonychidae and Trochanteriidae [50]. From diverse representatives of Salticidae only one single genus is known to lack claw tufts [59], although there are much more ground-dwelling species within this family. This may indicate that either costs associated with adhesive setae are rather low, or that, once evolved, these are highly conservative in some lineages, limiting a secondary loss.

In Thomisidae a reduction of adhesive scopulae (low number of ‘simple’ tapered setae with spatulae small or lacking, Fig. 7K) is widespread, although most of these spiders are plant-dwelling [4] and capable of overpowering oversized prey [60]. Interestingly, scopulae are more developed in the basic thomisid groups of the *Borboropactus* and the *Stephanopis* clade, mainly found on the ground and on tree bark. This leads to the hypothesis that some derived thomisids specialized in ambushing on flowers and leaves of herbal plants, which often feature conical cells and crystalline wax coverage [61], producing a small scale roughness where spatulae loose their efficiency [19]. In the following there may have been a trend of reduction of both seta and spatula width in those specialists. Adaptation effects may be indicated by the high variance of spatula sizes within this family (Fig. 5).

The hypothesis that adhesive setae are costly not only explains why they repeatedly got lost during evolution, but also why evolution favoured either adhesive setae or using silk, and why both together occur only rarely.

### Conclusions and Outlook

The widespread distribution of adhesive setae among spiders reflects their important ecological role. They provide a reusable adhesive alternative to using silk for prey capture and locomotion purposes. The ecological success of adhesive setae is reflected by their convergent evolution in several spider lineages. Morphological variations, reflecting habitat or prey specialisation (i.e. Thomisidae, Palpimanidae), and the adhesion-related evolutionary processes among Desidae and Dictynidae are worthy of more detailed studies in the future. Two important questions regarding the evolution of adhesive setae should also be answered in further experimental studies: (1) Which costs are associated with hairy attachment devices and (2) are there trade-offs between adhesive setae and other attachment devices, such as claws or adhesive silk.

## Supporting Information

Figure S1 **Table of surveyed material.**
(DOC)

Figure S2 **Evolution of setal types in the Araneomorphae.** A. Tree combining character traces from Ancestral State Analysis on dominant setal type of tarsus or pretarsus, if (false) claw tuft present. Blue shades indicate the highly anisotropic structured, but spatula lacking ‘frictional’ setae (FS). Yellow and red shades mark the adhesive setae of different types (AS). Purple lines indicate the serrated bristles (SB), being setal adaptations of the derived web building taxa. Setal type abbreviations are listed in detail in Fig. S1. B. Hypothesized evolutionary steps on setal morphology. The upper lineages assume independent evolution of adhesive pads in the pretarsus, whereas, in the lower ones, scopulae evolved first, then extended to the pretarsus, followed by claw tuft formation. Assumptions are based on the morphological comparison of scopula and claw tuft setae.
(PDF)

Figure S3 **Character states recorded for convergently evolved adhesive setae in the Araneae.**
(DOC)
