# Supplementary material for: The Great Silk Alternative: Multiple Co-Evolution of Web Loss and Sticky Hairs in Spiders
Source: PLoS One. 2013 May 1;8(5):e62682. doi: 10.1371/journal.pone.0062682 (PMC3641104; doi:10.1371/journal.pone.0062682)
Supplement: Figure S1 — Table of surveyed material. (DOC) [file pone.0062682.s001.doc]

S1. Data on the species included in the survey.

| **Family** | **Species number^1^** | **Species** | **adhesive pads^2^** | **dominant setal type^3^** | **foraging guild^4^** | **image references** | **further references** |
| --- | --- | --- | --- | --- | --- | --- | --- |
| Actinopodidae | 40 | *Missulena* sp. Walckenaer 1805 | - | - | fh | this study |  |
| Agelenidae | 1152 | *Malthonica ferruginea* Panzer 1804 | - | FS-II | wb | this study |  |
|  |  | *Tegenaria atrica* Koch 1843 | - | FS-II | wb | Coddington, 2010 |  |
|  |  | *Textrix denticulata* Olivier 1789 | - | FS-II | wb | Coddington, 2010 |  |
| Amaurobiidae | 285 | *Amaurobius fenestralis* Ström 1768 | - | FS-II | wb | this study |  |
|  |  | *Callevopsis striata* Tullgren 1902 | - | FS-II | wb | Benjamin & Hormiga, 2010 |  |
|  |  | *Callobius bennetti* Blackwall 1846 | - | FS-II | wb | Benjamin & Hormiga, 2010 |  |
|  |  | *Pimus napa* Leech 1972 | - | FS-II | wb | Ramírez, 2010 |  |
|  |  | *Retiro* sp. Mello-Leitao 1915 | - | FS-II | wb | Benjamin & Hormiga, 2010 |  |
|  |  | *Storenosoma* sp. Hogg 1900 | - | FS-II | wb | Benjamin & Hormiga, 2010 |  |
| Ammoxenidae | 18 | *Ammoxenus amphalodes* Dippenaar & Meyer 1980 | sc + ct | AS-II b / AS-II b-e | fh | Ramírez, 2004 | Joqué & Dippenaar-Schoeman, 2007 |
|  |  | *A. coccineus* Simon 1893 | sc + ct | AS-II b /  AS-II b-e | fh | this study |  |
| Amphinectidae | 159 | *Amphinecta pika* Forster & Wilton 1973 | - | - | fh | Griswold, 2006 |  |
|  |  | *Calacadia dentifera* Tullgren 1902 | - | FS-I | wb | Ramírez, 2004 |  |
|  |  | *Mamoea rufa* Berland 1931 | - | FS-II | fh | Griswold, 2006 |  |
|  |  | *Marplesia pohara* Forster & Wilton 1973 | - | FS-I | wb | Griswold, 2005 |  |
|  |  | *Metaltella simony* Keyserling 1878 | - | FS-II | wb | Griswold, 2005 |  |
|  |  | *Tasmarubrius truncus* Davies 1998 | - | FS-I | wb | Griswold, 2007 |  |
| Anapidae | 150 | *Elanapis aisen* Platnick & Forster 1989 | - | - / SB | wb | Lopardo, 2006 |  |
| Antrodiaetidae | 33 |  | - | n. a. | fh | no data | Joqué & Dippenaar-Schoeman, 2007 |
| Anyphaenidae | 517 | *Amaurobioides africana* Hewitt 1970 | ct | FS-I - II / AS-II a-e | fh | Ramírez, 2002 |  |
|  |  | *Anyphaena accentuata* Walckenaer 1802 | sc + ct | AS-II b / AS-II a-e | fh | Wolff & Gorb, 2012c |  |
|  |  | *Gayenna americana* Nicolet 1849 | sc + ct | AS-II b / AS-II a-e | fh | Ramírez, 2002 |  |
|  |  | *Malenella nana* Ramírez 1995 | ct | FS-I – II / AS-II a | fh | Ramírez, 2002 |  |
|  |  | *Tomopisthes varius* Simon 1884 | sc + ct | AS-II b / AS-II a-e | fh | Ramírez, 1995 |  |
|  |  | *Xiruana gracilipes* Keyserling 1891 | sc + ct | AS-II b / AS-II a-e | fh | Ramírez, 2002 |  |
| Araneidae | 3031 | *Araneus diadematus* Clerck 1757 | - | - / SB | wb | Scharff, 2010 | Foelix, 1970 |
| Archaeidae | 62 | *Afrarchaea woodae* Lotz 2006 | sc | AS-II b / FS-II | fh | Griswold, 2010 |  |
|  |  | *Austrarchaea nodosa* Forster 1956 | sc | AS-II b / FS-II | fh | Griswold, 2010 | Forster & Platnick, 1984 |
|  |  | *Eriauchenius workmani* Cambridge 1881 | sc | AS-II b / FS-II | fh | Ramírez, 2002 |  |
| Atypidae | 49 | *Calommata tibialis* Fourie et al. 2011 | - | - | fh | Fourie et al. 2011 | Joqué & Dippenaar-Schoeman, 2007 |
| Austrochilidae | 9 | *Thaida peculiaris* Karsch 1880 | - | FS-II / SB | wb | Ramírez, 2010 |  |
| Barychelidae | 307 | *Encyocrypta* sp. Simon 1889 | sc + ct | AS-II b | fh | this study |  |
| Caponiidae | 84 | *Nops* sp. MacLeay 1839 | - | FS-I - II | fh | Ramírez, 2010 |  |
|  |  | *N. largus* Chickering 1967 | - | FS-I - II | fh | this study |  |
| Cithaeronidae | 7 | *Cithaeron delimbatus* Strand 1906 | sc + ct | AS-II b | fh | Ramírez, 2002 |  |
| Clubionidae | 581 | *Carteronius* sp. Simon 1897 | ct | FS-I / AS-III | fh | Ramírez, 2002 |  |
|  |  | *Clubiona caerulescens* L. Koch 1867 | sc + ct | AS-II b / AS-III | fh | Wolff and Gorb, 2012c |  |
|  |  | *C. comta* Koch 1837 | sc + ct | AS-II b / AS-III | fh | this study |  |
|  |  | *C. frisia* Wunderlich & Schuett 1995 | sc + ct | AS-II b / AS-III | fh | this study |  |
|  |  | *C. lutescens* Westring 1853 | sc + ct | AS-II b / AS-III | fh | this study |  |
|  |  | *C. neglecta* Cambridge 1862 | sc + ct | AS-II b / AS-III | fh | this study |  |
|  |  | *C. pallidula* Clerck 1757 | sc + ct | AS-II b / AS-III | fh | this study | Ramírez, 2002 |
|  |  | *C. phragmitis* Koch 1843 | sc + ct | AS-II b / AS-III | fh | this study |  |
|  |  | *C. reclusa* Cambridge 1863 | sc + ct | AS-II b / AS-III | fh | this study |  |
|  |  | *C. terrestris* Westring 1851 | sc + ct | AS-II b / AS-III | fh | this study |  |
|  |  | *Elaver texana* Gertsch 1933 | sc + ct | AS-II b / AS-III | fh | Davila, 2003 |  |
|  |  | *Elaver* sp. Cambridge 1898 | sc + ct | AS-II b / AS-III | fh | Ramírez, 2002 |  |
| Corinnidae | 1014 | *Brachyphaea* sp. Simon 1895 | sc + ct | AS-II b | fh | Ramírez, 2002 |  |
|  |  | *Castianeira trilineata* Hentz 1847 | sc + ct | AS-II b | fh | Ramírez, 2002 |  |
|  |  | *Copa flavoplumosa* Simon 1886 | sc + ct | AS-II b | fh | Ramírez, 2002 |  |
|  |  | *Corinna bulbula* Cambridge 1899 | sc + ct | AS-II b | fh | Ramírez, 2002 |  |
|  |  | *Drassinella gertschi* Platnick & Ubick, 1989 | sc + ct | AS-II b | fh | Ramírez, 2002 | Ubick & Vetter, 2005 |
|  |  | *Falconina gracilis* Keyserling 1891 | sc + ct | AS-II b | fh | Ramírez, 2009 |  |
|  |  | *Hortipes* sp. Bosselaers & Ledoux, 1998 | ct | FS-I / AS-II b | fh | Ramírez, 2006 | Bosselaers & Joqué, 2000 |
|  |  | *Lessertina mutica* Lawrence 1942 | sc + ct | AS-II b | fh | Ramírez, 2010 |  |
|  |  | *Mandaneta sudana* Karsch 1880 | sc + ct | AS-II b | fh | Ramírez, 2002 |  |
|  |  | *Medmassa proxima* Lessert 1923 | sc + ct | AS-II b | fh | Ramírez, 2010 |  |
|  |  | *Meriola barrosi* Mello-Leitao 1951 | sc + ct | AS-II b / AS-II b-e | fh | Ramírez, 2010 |  |
|  |  | *Orthobula* sp. Simon 1897 | sc + ct | AS-II b | fh | Ramírez, 2002 |  |
|  |  | *Paccius* sp. Simon 1898 | sc + ct | AS-II b / AS-III-e | fh | Ramírez, 2010 |  |
|  |  | *Paradiestus penicillatus* Mello-Leitao 1939 | sc + ct | AS-II b | fh | Ramírez, 2010 |  |
|  |  | *Phrurolithus festivus* Koch 1835 | ct | FS-I / AS-II b | fh | this study | Ramírez, 2010 |
|  |  | *Phrurotimpus alarius* Hentz 1847 | ct | FS-I / AS-II b | fh | Ramírez, 2010 |  |
|  |  | *Trachelas mexicanus* Banks 1898 | sc + ct | AS-II b / AS-III | fh | Ramírez, 2010 |  |
|  |  | *Trachelopachys ammobates* Platnick & Rocha 1995 | sc + ct | AS-II b | fh | Ramírez, 2002 |  |
| Ctenidae | 468 | *Amauropelma trueloves* Raven & Stumkat 2001 | ct | FS-II / AS-II b | fh | Griswold, 2006 |  |
|  |  | *Anahita* sp. Karsch 1879 | sc + ct | AS-II b | fh | Griswold, 2008 |  |
|  |  | *Ancylometes bogotensis* Keyserling 1877 | sc | AS-II b / FS-II | fh | Griswold, 2008 |  |
|  |  | *Caloctenus oxapampa* Silva 2004 | sc + ct | AS-II b | fh | Griswold, 2005 |  |
|  |  | *Ctenus* sp. Walckenaer 1805 | sc + ct | n. a. | fh | Ramírez, 2002 |  |
|  |  | *C. dubius* Walckenaer 1805 | sc + ct | AS-II b | fh | Griswold, 2005 |  |
|  |  | *C. medius* Keyserling 1891 | sc + ct | AS-II b | fh | this study |  |
|  |  | *C. yaeyamensis* Yoshida 1998 | sc + ct | AS-II b | fh | Griswold, 2005 |  |
|  |  | *Cupiennius coccineus* Cambridge 1901 | sc + ct | AS-II b | fh | Lapinski, 2009 |  |
|  |  | *C. salei* Keyserling 1877 | sc + ct | AS-I a + II b /  AS-II b | fh | this study | Wolff & Gorb, 2012c |
|  |  | *Gephyroctenus* sp. Mello-Leitao 1936 | ct | n. a. | fh | Davila, 2003 |  |
|  |  | *Nothroctenus fuxico* Dias & Brescovit 2004 | sc + ct | n. a. | fh | Dias & Brescovit, 2004 |  |
|  |  | *Phoneutria boliviensis* Cambridge 1897 | sc + ct | AS-II b | fh | Lapinski, 2009 |  |
|  |  | *Vulsor isaloensis* Ono 1993 | sc + ct | AS-II b | fh | Griswold, 2005 |  |
|  |  | *Xenoctenus* sp. Mello-Leitao 1938 | sc | n. a. | fh | Davila, 2003 |  |
| Ctenizidae | 128 |  | - | n. a. | fh | no data | Joqué & Dippenaar-Schoeman, 2007 |
| Cybaeidae | 177 | *Argyroneta aquatica* Clerck 1757 | - | FS-II / SB | wb | Griswold, 2007 |  |
|  |  | *Cybaeus* sp. Koch 1868 | - | - | wb | Coddington, 2010 |  |
| Cyatholipidae | 58 | *Toddiana* sp. Forster 1988 | - | SB | wb | Griswold, 2010 |  |
| Cycloctenidae | 36 | *Cycloctenus nelsonensis* Forster 1979 | - | FS-I | fh | Griswold, 2006 |  |
|  |  | *Toxopsiella minuta* Forster 1964 | - | FS-I | fh | Ramírez, 2010 |  |
| Cyrtaucheniidae | 101 | *Promyrmekiaphila clathrata* Simon 1891 | sc | AS-I a / FS-I | fh | Bond, 2010 |  |
| Deinopidae | 57 |  | - | n. a. | wb | no data | Joqué & Dippenaar-Schoeman, 2007 |
| Desidae | 181 | *Badumna longinqua* Koch 1867 | - | FS-II | wb | Griswold, 2006 | Forster, 1970 |
|  |  | *Desis* spp. Walckenaer 1837 | sc | n. a. | fh | no data | Forster, 1970 |
|  |  | *Goyenia* spp. Forster 1970 | ct | FS-II / AS-II b-e | fh | Griswold, 2006 | Forster, 1970 |
|  |  | *Helsonia plata* Forster 1970 | ct | n. a. | fh | Forster, 1970 | Forster, 1970 |
|  |  | *Manawa solitaria* Forster 1970 | ct | n. a. | fh | Forster, 1970 | Forster, 1970 |
|  |  | *Otagoa nova* Forster 1970 | - | FS-I | wb | Coddington, 2010 |  |
|  |  | *Paramatachia* spp. Dalmas 1918 | sc | n. a. | fh | no data | Forster, 1970 |
|  |  | *Rapua australis* Forster 1970 | ct | n. a. | fh | Forster, 1970 | Forster, 1970 |
|  |  | *Toxops* sp. Forster 1964 | ct | FS-I / AS-II b | fh | Griswold, 2005 | Forster, 1970 |
| Dictynidae | 571 | *Blabomma guttatum* Chamberlin & Ivie 1937 | - | FS-I | wb | Benjamin & Hormiga, 2010 |  |
|  |  | *Dictyna arundinacea* Linn. 1758 | - | FS-II | wb | Ramírez, 2004 |  |
|  |  | *Emblyna sublata* Hentz 1850 | - | FS-II | wb | Benjamin & Hormiga, 2010 |  |
|  |  | *Mallos dugesi* Banks 1886 | - | FS-II | wb | Benjamin & Hormiga, 2010 |  |
|  |  | *Mexitlia trivittata* Banks 1901 | - | FS-II | wb | Benjamin & Hormiga, 2010 |  |
|  |  | *Paradictyna ilamia* Forster 1970 | ct | n. a. | fh | Forster, 1970 | Forster, 1970 |
|  |  | *Viridictyna* spp. Forster 1970 | ct | n. a. | fh | Forster, 1970 | Forster, 1970 |
| Diguetidae | 15 | *Diguetia catamarquensis* Mello-Leitao 1941 | - | SB | wb | Ramírez, 2010 |  |
| Dipluridae | 180 |  | - | n. a. | wb | no data | Joqué & Dippenaar-Schoeman, 2007 |
| Drymusidae | 15 | *Drymusa rengan* Labarque & Ramírez 2007 | - | FS-II | wb | Ramírez, 2010 |  |
| Dysderidae | 526 | *Dysdera crocata* Koch 1838 | ct | FS-II / AS-I a | fh | this study | Griswold, 2007 |
|  |  | *Harpactea hombergi* Bristowe 1939 | - | FS-I | fh | this study |  |
| Eresidae | 95 | *Stegodyphus* sp. Simon 1873 | - | FS-II | wb | Griswold, 2010 |  |
| Filistatidae | 113 | *Filistata insidiatrix* Forsskål 1775 | - | FS-II | fh | Ramírez, 2002 |  |
|  |  | *Kukulcania hibernalis* Hentz 1842 | - | FS-II | fh | Ramírez, 2010 |  |
|  |  | *Pikelinia tambilloi* Mello-Leitao 1941 | - | FS-II | fh | Ramírez, 2010 |  |
|  |  | *Pritha nana* Simon 1876 | - | FS-II | fh | Ramírez, 2002 |  |
| Gallieniellidae | 57 | *Austrachelas* sp. Lawrence 1938 | sc + f-ct | AS-II b / AS-II b-e | fh | Ramírez, 2010 | Haddad et al., 2009 |
|  |  | *Drassodella vasivulva* Tucker 1923 | f-ct | n. a. / AS-II b-e | fh | Warui & Joqué, 2002 |  |
|  |  | *Galianoella leucostigma* Mello-Leitao 1941 | - | FS-I | fh | Ramírez, 2007 |  |
|  |  | *Legendrena perinet* Platnick 1984 | - | FS-I | fh | Ramírez, 2002 |  |
|  |  | *Meedo houstoni* Main 1987 | - | FS-I | fh | Ramírez, 2002 |  |
| Gnaphosidae | 2134 | *Apodrassodes quilpuensis* Simon 1902 | sc + f-ct | AS-II b | fh | Ramírez, 2006 |  |
|  |  | *Camillina calel* Platnick & Shadab 1982 | sc | FS-II / AS-II b | fh | Ramírez, 2006 |  |
|  |  | *Drassyllus praeficus* Koch 1866 | sc | AS-II b / FS-I | fh | this study | Wolff and Gorb, 2012c |
|  |  | *Drassodes lapidosus* Walckenaer 1802 | sc + f-ct | AS-II b / AS-II b-e | fh | this study |  |
|  |  | *Eilica amambay* Platnick 1985 | sc + f-ct | FS-I / AS-II b | fh | Ramírez, 2006 |  |
|  |  | *Gnaphosa* sp. Latreille 1804 | sc + f-ct | AS-II b | fh | this study |  |
|  |  | *G. sericata* Koch 1866 | sc | AS-II b / FS-II | fh | Ramírez, 2010 |  |
|  |  | *Haplodrassus signifer* Koch 1839 | sc + f-ct | AS-II b | fh | this study |  |
|  |  | *Micaria formicaria* Sundevall 1831 | sc + f-ct | AS-II b | fh | this study |  |
|  |  | *M. fulgens* Walckenaer 1802 | sc + f-ct | AS-II b / AS-II b-e | fh | Ramírez, 2002 |  |
|  |  | *M. pulicaria* Sundevall 1831 | sc + f-ct | AS-II b | fh | this study |  |
|  |  | *Vectius niger* Simon 1880 | sc | AS-II b / - | fh | Ramírez, 2006 |  |
|  |  | *Xenoplectus* sp. Schiapelli & Gerschman 1958 | sc | AS-II b / FS-II | fh | Ramírez, 2010 |  |
|  |  | *Zelotes clivicola* Koch 1870 | sc + f-ct | AS-II b | fh | this study |  |
|  |  | *Z. subterraneus* Koch 1833 | sc + f-ct | AS-II b | fh | Wolff and Gorb, 2012c |  |
| Gradungulidae | 16 | *Gradungula sorenseni* Forster 1955 | - | - | wb | Ramírez, 2010 |  |
|  |  | *Macrogradungula moonya* Gray 1987 | - | - | fh | Ramírez, 2010 |  |
| Hahniidae | 247 | *Cybaeolus rastellus* Roth 1967 | - | FS-I | wb | Benjamin & Hormiga, 2010 |  |
| Hersiliidae | 176 | *Ypypuera crucifera* Vellard 1924 | - | FS-I | wb | Ramírez, 2010 |  |
| Hexathelidae | 105 |  | - | n. a. | wb | no data | Joqué & Dippenaar-Schoeman, 2007 |
| Holarchaeidae | 2 | *Holarchaea* sp. Forster 1955 | - | SB | fh (?) | Griswold, 2007 | Forster & Platnick, 1984 |
| Homalonychiidae | 3 | *Homalonychus selenopoides* Marx 1891 | ct | - / AS-I a | fh | this study |  |
|  |  | *H. theologus* Chamberlin 1924 | ct | - / AS-I a | fh | Ramírez, 2010 |  |
| Huttoniidae | 1 | *Huttonia palpimanoides* Cambridge 1879 | sc | FS-I / AS-II b | fh | Forster & Platnick, 1984 |  |
| Hypochilidae | 12 | *Hypochilus pococki* Platnick 1987 | - | - | wb | Ramírez, 2010 |  |
| Idiopidae | 314 | *Arbanitis* sp. Koch 1874 | sc | - / AS-I a | fh | this study |  |
|  |  | *Idiops pylorus* Schwendinger 1991 | - | - | fh | Foelix et al., 2010 |  |
|  |  | *Neocteniza* sp. Pocock 1895 | sc | - / AS-I a | fh | this study |  |
| Lamponidae | 192 | *Lampona cylindrata* Koch 1866 | sc + f-ct | AS-II b | fh | Ramírez, 2002 |  |
|  |  | *Lamponella brookfield* Platnick 2000 | sc + f-ct | AS-II b | fh | Ramírez, 2010 |  |
|  |  | *Pseudolampona emmett* Platnick 2000 | sc + f-ct | AS-II b | fh | Ramírez, 2010 |  |
| Leptonetidae | 271 | *Archoleptoneta schusteri* Gertsch 1974 | - | FS-II | wb | Griswold, 2006 |  |
|  |  | *Leptoneta infuscata* Simon 1872 | - | FS-II | wb | Griswold, 2007 |  |
| Linyphiidae | 4419 | *Drapetisca socialis* Sundevall 1833 | - | SB | wb | this study |  |
|  |  | *Dubiaranea aysenensis* Nicolet 1849 | - | SB | wb | Benjamin & Hormiga, 2010 |  |
|  |  | *Microlinyphia* sp. Gerhardt 1928 | - | SB | wb | Benjamin & Hormiga, 2010 |  |
|  |  | *Stemonyphantes lineatus* Linn. 1758 | - | SB | wb | Benjamin & Hormiga, 2010 |  |
| Liocranidae | 186 | *Agroeca brunnea* Blackwall 1833 | sc | AS-II b / FS-II | fh | Ramírez, 2002 | Wolff & Gorb, 2012c |
|  |  | *Apostenus californicus* Ubick & Vetter, 2005 | sc + ct | AS-II b / AS-II b-e | fh | Ramírez, 2002 | Ubick & Vetter, 2005 |
|  |  | *Jacaena* sp. Thorell 1897 | - | FS-I | fh | Ramírez, 2010 |  |
|  |  | *Liocranum rupicola* Walckenaer 1830 | sc | AS-II b / FS-I | fh | this study | Ramírez, 2002; Ubick & Vetter, 2005 |
| Liphistiidae | 90 | *Heptathela* sp. Kishida 1923 | - | - | fh | this study |  |
|  |  | *Liphistius desultor* Schiödte 1830 | - | - | fh | Foelix et al., 2010 |  |
|  |  | *Liphistius endau* Sedgwick & Platnick 1987 | - | - | fh | Foelix et al., 2010 |  |
| Lycosidae | 2388 | *Aglaoctenus lagotis* Holmberg 1876 | sc | AS-II b / FS-II | fh | Ramírez, 2010 |  |
|  |  | *Alopecosa* sp. Clerck 1757 | sc | AS-II b / FS-I | fh | this study |  |
|  |  | *Aulonia albimana* Walckenaer 1805 | - | FS-II | wb | this study |  |
|  |  | *Hogna coloradensis* Banks 1894 | sc | AS-II b / FS-I | fh | Sierwald, 2008 |  |
|  |  | *Rabidosa hentzi* Banks 1904 | sc + f-ct | AS-II b | fh | Miller et. al, 1988 |  |
|  |  | *R. punctulata* Hentz 1844 | sc | AS-II b / FS-I | fh | this study | Rovner, 1978 |
|  |  | *Pardosa wagleri* Hahn 1822 | sc | AS-II b / FS-I | fh | this study |  |
|  |  | *Piratula hygrophila* Thorell 1872 | sc | AS-II b / FS-I | fh | this study |  |
|  |  | *Rabidosa rabida* Walckenaer 1837 | sc | AS-II b / FS-II | fh | this study | Miller et. al, 1988; Rovner, 1978; Sierwald, 2008 |
|  |  | *Schizocosa* sp. Chamberlin 1904 | sc | AS-II b / FS-II | fh | this study |  |
|  |  | *Sosippus* sp. Simon 1888 | sc | AS-II b / FS-II | wb | this study | Rovner, 1978 |
|  |  | *Trabea paradoxa* Simon 1876 | sc + f-ct | AS-II b | fh | this study |  |
|  |  | *Xerolycosa nemoralis* Westring 1861 | sc | AS-II b / FS-II | fh | this study |  |
| Malkaridae | 11 | *Carathea parawea* Moran 1986 | - | SB | fh |  |  |
| Mecicobothriidae | 9 |  | - | n. a. | wb | no data | Joqué & Dippenaar-Schoeman, 2007 |
| Mecysmaucheniidae | 25 | *Mecysmauchenius segmentatus* Simon 1884 | - | FS-I | fh | Griswold, 2010 |  |
| Micropholcommatidae | 66 | *Textricella luteola* Hickman 1945 | - | SB | wb | Ramírez, 2010 |  |
| Migidae | 91 | indet. | - | - | fh | this study |  |
| Mimetidae | 156 | *Ero aphana* Walckenaer 1802 | - | SB | fh | Scharff, 2007 |  |
| Miturgidae | 359 | *Cheiracanthium erraticum* Walckenaer 1802 | sc + ct | AS-II b | fh | this study |  |
|  |  | *C. punctorium* Villers 1789 | sc + ct | AS-II b | fh | this study | Ramírez, 2002 |
|  |  | *Cheiramiona* sp. Lotz & Dippenaar-Schoeman 1999 | ct | FS-II / AS-II b | fh | Ramírez, 2002 |  |
|  |  | *Eutichurus lizeri* Mello-Leitao 1938 | sc + ct | AS-II b | fh | Ramírez, 2002 |  |
|  |  | *Mituliodon tarantulinus* Koch 1873 | sc + ct | AS-II b | fh | Ramírez, 2010 |  |
|  |  | *Strotarchus piscatorius* Hentz 1847 | sc | AS-II b / FS-II | fh | Ramírez, 2010 |  |
|  |  | *Syspira eclectica* Chamberlin 1924 | sc + f-ct | AS-II b | fh | Ramírez, 2002 |  |
|  |  | *Teminius insularis* Lucas 1957 | sc + f-ct | AS-II b | fh | Ramírez, 2002 |  |
|  |  | *Uliodon* sp. Koch 1873 | sc + f-ct | AS-I a | fh | Ramírez, 2010 |  |
| Mysmenidae | 123 | *Mysmena tasmaniae* Hickman 1979 | - | - / SB | wb | Ramírez, 2010 |  |
| Nemesiidae | 356 | *Calisoga* sp. Chamberlin 1937 | sc + f-ct | AS-I a | fh | Bond, 2010 |  |
|  |  | *Ixamatus* sp. Simon 1887 | sc | AS-I a / - | fh | this study |  |
|  |  | *Nemesia* sp. Audouin 1826 | sc + f-ct | AS-I a | fh | this study |  |
| Nesticidae | 209 | *Eidmannella pallida* Emerton 1857 | - | - / SB | wb | Coddington, 2010 |  |
|  |  | *Nesticus archeri* Gertsch 1984 | - | - / SB | wb | Coddington, 2010 |  |
| Nicodamidae | 29 | *Novodamus nodatus* Karsch 1878 | - | - / SB | wb | Scharff, 2007 |  |
| Ochyroceratidae | 161 | *Ochyrocera* sp. | - | FS-II | wb | Griswold, 2008 |  |
| Oecobiidae | 110 | *Uroctea durandi* Latreille 1809 | - | - | wb | this study |  |
| Oonopidae | 1016 | *Birabenella homonota* Grismado 2010 | ct | FS-II / AS-II b | fh | Grismado, 2010 |  |
|  |  | *Camptoscaphiella paquini* Baehr & Ubick 2010 | ct | FS-II / AS-II b-e | fh | Baehr & Ubick, 2010 |  |
|  |  | *Cortestina thaleri* Knoflach 2009 | ct | FS-II / AS-II b | fh | Knoflach et al., 2009 |  |
|  |  | *Heteroonops castellus* Chickering 1971 | ct | FS-II / AS-II b-e | fh | Platnick & Dupérré, 2009 |  |
|  |  | *Malagiella ranomafana* Ubick & Griswold 2011 | ct | FS-II / AS-II b | fh | Ubick & Griswold, 2011 |  |
|  |  | *Melchisedec thevenot* Fannes 2010 | ct | FS-II / AS-II b | fh | Fannes, 2010 |  |
|  |  | *Opopaea* sp. Simon 1891 | ct | FS-II / AS-II b-e | fh | Griswold, 2007 |  |
|  |  | *Oonops pulcher* Templeton 1835 | ct | FS-II / AS-II b-e | fh | Platnick & Dupérré, 2009 |  |
|  |  | *Unicorn sikus* Reyes 2010 | ct | FS-II / AS-II b-e | fh | Reyes et al., 2010 |  |
| Orsolobidae | 184 | *Afrilobus capensis* Griswold & Platnick 1987 | ct | n. a. / AS-II b | fh | Griswold & Platnick, 1987 |  |
|  |  | *Azanialobus lawrencei* Griswold & Platnick 1987 | ct | FS-II / AS-II b | fh | Griswold & Platnick, 1987 |  |
|  |  | *Falklandia rumbolli* Schiapelli & Gerschman 1974 | ct | FS-II / AS-II b | fh | this study |  |
|  |  | *Hickmanolobus linnaei* Baehr & Smith 2008 | ct | FS-II / AS-II b | fh | Baehr & Smith, 2008 |  |
|  |  | *Losdolobus opytapora* Brescovit 2004 | ct | n. a. / AS-II b | fh | Brescovit et al., 2004 |  |
|  |  | *Orsolobus pucara* Forster & Platnick 1985 | ct | FS-II / AS-II b | fh | Izquierdo & Labarque, 2010 | Ramírez, 2010 |
|  |  | *Osornolobus* sp. Forster & Platnick 1985 | ct | FS-II / AS-I a | fh | Ramírez, 2010 |  |
| Oxyopidae | 433 | *Oxyopes heterophthalmus* Latreille 1804 | - | FS-II | fh | Wolff & Gorb, 2012c | Ramírez, 2010 |
| Palpimanidae | 131 | *Otiothops birabeni* Mello-Letao 1945 | sc + ct | AS-II b / AS-II b-e | fh | Ramírez, 2010 |  |
|  |  | *Otiothops macleayi* Banks 1929 | sc + ct | AS-II b / AS-II b-e | fh | Forster & Platnick, 1984 |  |
|  |  | *Palpimanus gibbulus* Dufour 1820 | sc | AS-II b / FS-II | fh | this study |  |
|  |  | *Palpimanus transvaalicus* Simon 1893 | sc + ct | AS-II b | fh | Ramírez, 2010 |  |
|  |  | *Sarascelis chaperi* Simon 1887 | sc + ct | AS-II b / AS-III-e | fh | Ramírez, 2010 |  |
|  |  | *Scelidocteus* sp. Simon 1907 | sc + ct | AS-II b | fh | Forster & Platnick, 1984 |  |
| Pararchaeidae | 35 | *Pararchaea* sp. Forster 1955 | - | SB | fh | Griswold, 2010 |  |
| Paratropidae | 8 |  | ct | n. a. | fh | no data | Joqué & Dippenaar-Schoeman, 2007 |
| Periegopidae | 2 | *Periegops suteri* Urquhart 1892 | - | - | fh | Ramírez, 2010 |  |
| Philodromidae | 538 | *Ebo mexicanus* Banks 1898 | sc + ct | AS-II b | fh | Ramírez, 2010 |  |
|  |  | *Halodromus patellaris* Wunderlich 1987 | sc + ct | AS-II b / AS-II b-e | fh | Muster, 2009 |  |
|  |  | *Petrichus* sp. Simon 1886 | sc + ct | AS-II b | fh | Ramírez, 2010 |  |
|  |  | *Philodromus albidus* Kulczynski 1911 | sc + ct | AS-II b / AS-II b-e | fh | this study |  |
|  |  | *P. aureolus* Clerck 1757 | sc + ct | AS-II b / AS-II b-e | fh | this study | Foelix & Chu-Wang, 1975 |
|  |  | *P. cespitum* Walckenaer 1802 | sc + ct | AS-II b / AS-II b-e | fh | Wolff & Gorb, 2012c |  |
|  |  | *P. collinus* Koch 1835 | sc + ct | AS-II b / AS-II b-e | fh | this study |  |
|  |  | *P. dispar* Walckenaer 1826 | sc + ct | AS-II b / AS-II b-e | fh | Wolff & Gorb, 2012b |  |
|  |  | *P. fallax* Sundevall 1833 | sc + ct | AS-II b | fh | this study |  |
|  |  | *Tibellus oblongus* Walckenaer 1802 | sc + ct | AS-II b / AS-II b-e | fh | Wolff & Gorb, 2012c | Ramírez, 2010 |
|  |  | *Thanatus formicinus* Clerck 1757 | sc + ct | AS-II b / AS-II b-e | fh | Wolff & Gorb, 2012c |  |
| Pholcidae | 1288 | *Holocnemus pluchei* Scopoli 1763 | - | FS-II | wb | Griswold, 2007 |  |
| Phyxelididae | 64 | *Vidole capensis* Pocock 1900 | - | FS-II | wb | Griswold, 2006 |  |
|  |  | *Vytfutia* sp. Deeleman-Reinhold 1986 | - | FS-II | wb | Griswold, 2008 |  |
| Pimoidae | 37 | *Pimoa altioculata* Keyserling 1886 | - | - / SB | wb | Benjamin & Hormiga, 2010 |  |
| Pisauridae | 331 | *Dolomedes fimbriatus* Clerck 1757 | sc | AS-II b / FS-II | fh | this study |  |
|  |  | *D. tenebrosus* Hentz 1844 | sc | AS-II b / FS-II | fh | Ramírez, 2010 |  |
|  |  | *Thalassius spinosissimus* Karsch 1879 | - | - | fh | Sierwald, 2008 |  |
| Plectreuridae | 31 | *Plectreurys tristis* Simon 1893 | - | FS-II | wb | Ramírez, 2010 |  |
| Prodidomidae | 303 | *Chilongius palmas* Platnick et al. 2005 | ct | FS-II / AS-II b | fh | Platnick et al., 2005 |  |
|  |  | *Lygromma* sp. Simon 1893 | ct | FS-II / AS-II b | fh | Ramírez, 2010 |  |
|  |  | *Moreno chivato* Platnick et al. 2005 | ct | FS-II / AS-II b | fh | Platnick et al., 2005 |  |
|  |  | *Neozimiris pubescens* Banks 1898 | ct | FS-II / AS-II b | fh | Ramírez, 2010 |  |
|  |  | cf. *Tricongius* sp. Simon 1893 | sc + ct | AS-II b | fh | Ramírez, 2010 |  |
|  |  | *Zimiris doriai* Simon 1882 | ct | FS-II / AS-II b | fh | Platnick & Penney, 2004 |  |
| Psechridae | 30 | *Psechrus argentatus* Doleschall 1859 | ct | FS-II / AS-II b | wb | Ramírez, 2010 |  |
|  |  | *P. clavis* Bayer 2012 | ct | FS-II / AS-II b | wb | this study |  |
|  |  | *P. luangprabang* Jäger 2007 | ct | FS-II / AS-II b | wb | this study |  |
|  |  | *Fecenia cylindrata* Thorell 1895 | ct | FS-II / AS-II b | wb | this study |  |
| Salticidae | 5468 | *Aelurillus andreevae* Nenilin 1984 | ct | n. a. | fh | Logunov, 1996 |  |
|  |  | *Asianellus festivus* Koch 1834 | sc + f-ct | AS-II b | fh | this study |  |
|  |  | *Cocalodes longicornis* Wanless 1982 | sc + ct | AS-II b | fh | Ramírez, 2010 |  |
|  |  | *Euophrys frontalis* Walckenaer 1802 | ct | FS-I / AS-II b | fh | this study |  |
|  |  | *Evarcha arcuata* Clerck 1757 | ct | FS-I / AS-II b | fh | Wolff & Gorb, 2012c | Kesel et al., 2003 |
|  |  | *E. falcata* Clerck 1757 | ct | FS-I / AS-II b | fh | this study | Ramírez, 2010 |
|  |  | *Freya* sp. Koch 1850 | f-ct | FS-I / AS-II b | fh | Ramírez, 2010 |  |
|  |  | *Hasarius adansoni* Audouin 1826 | f-ct | FS-I / AS-II b | fh | this study |  |
|  |  | *Heliophanus cupreus* Walckenaer 1802 | ct | FS-I / AS-II b | fh | Wolff & Gorb, 2012c |  |
|  |  | *H. flavipes* Hahn 1832 | ct | FS-I / AS-II b | fh | this study |  |
|  |  | *Hispo* sp. Simon 1886 | f-ct | FS-II / AS-II b | fh | Ramírez, 2010 |  |
|  |  | *Holcolaetis* sp. Simon 1886 | sc + ct | AS-II b | fh | Ramírez, 2010 |  |
|  |  | *Lyssomanes viridis* Walckenaer 1837 | ct | - / AS-II b | fh | Ramírez, 2010 |  |
|  |  | *Marpissa muscosa* Clerck 1757 | ct | - / AS-II b | fh | this study |  |
|  |  | *Menemerus bivittatus* Dufour 1831 | ct | FS-I / AS-II b | fh | Ramírez, 2010 |  |
|  |  | *Nannenus* sp. Simon 1902 | f-ct | FS-I / AS-I b | fh | Ramírez, 2010 |  |
|  |  | *Neon reticulatus* Blackwall 1853 | ct | FS-I / AS-II b | fh | this study |  |
|  |  | *Phiale* sp. Koch 1846 | ct | FS-I / AS-II b | fh | this study |  |
|  |  | *Phidippus audax* Hentz 1845 | sc + ct | AS-II b | fh | Hill, 1977 |  |
|  |  | *P. regius* Koch 1846 | sc + ct | AS-II b | fh | Foelix & Erb, 2011 |  |
|  |  | *Phlegra fasciata* Hahn 1826 | sc + f-ct | AS-II b | fh | this study |  |
|  |  | *Plexippus setipes* Karsch 1879 | ct | FS-I / AS-II b | fh | Moon & Park, 2009 |  |
|  |  | *Portia schultzi* Karsch 1878 | sc + ct | AS-II b | fh | Ramírez, 2010 | Foelix et al., 1984 |
|  |  | *Proszynskiana starobogatovi* Logunov 1996 | - | - | fh | Logunov, 1996 |  |
|  |  | *Pseudeuophrys lanigera* Simon 1871 | ct | FS-I / AS-II b | fh | this study |  |
|  |  | *Salticus scenicus* Clerck 1757 | ct | n. a. / AS-II b | fh | Roscoe & Walker, 1991 |  |
|  |  | *Sassacus papenhoei* Peckham & Peckham 1895 | ct | FS-II / AS-II b | fh | Hill, 1977 |  |
|  |  | *Spartaeus wildtrackii* Wanless 1987 | sc + ct | AS-II b | fh | Wanless, 1987 | Ramírez, 2010 |
|  |  | *Synageles venator* Lucas 1836 | ct | - / AS-II b | fh | this study |  |
| Scytodidae | 228 | *Scytodes globula* Nicolet 1849 | - | FS-II | wb | Ramírez, 2010 |  |
| Segestriidae | 118 | *Ariadna boesenbergi* Keyserling 1877 | - | FS-II | fh | Ramírez, 2010 |  |
| Selenopidae | 239 | *Anyphops* sp. Benoit 1968 | sc + ct | AS-II b | fh | Ramírez, 2010 |  |
|  |  | *Hovops* sp. Benoit 1968 | sc + ct | AS-II b | fh | Ramírez, 2010 |  |
|  |  | *Selenops debilis* Banks 1898 | sc + ct | AS-II b | fh | Ramírez, 2010 |  |
|  |  | *S. mexicanus* Keyserling 1880 | ct | FS-I / AS-II b | fh | this study |  |
| Senoculidae | 31 | *Senoculus purpureus* Simon 1880 | - | - | fh | Ramírez, 2010 |  |
| Sicariidae | 125 | *Loxosceles rufescens* Dufour 1820 | - | FS-II | fh | Ramírez, 2010 |  |
|  |  | *Sicarius* sp. Walckenaer 1847 | - | - | fh | this study |  |
|  |  | *S. rupestris* Holmberg 1881 | - | - | fh | Ramírez, 2010 |  |
| Sparassidae | 1123 | *Eusparassus* sp. Simon 1903 | sc + ct | AS-II b / AS-II c | fh | Ramírez, 2010 |  |
|  |  | *Heteropoda venatoria* Linn. 1767 | sc + ct | AS-II b | fh | this study | Ramírez, 2010 |
|  |  | *Micrommata ligurina* Koch 1845 | sc + ct | n. a. | fh | Rambla, 1990 |  |
|  |  | *M. virescens* Clerck 1757 | sc + ct | AS-II b / AS-II c | fh | Wolff & Gorb, 2012c |  |
|  |  | *Polybetes pythagoricus* Holmberg 1875 | sc + ct | AS-II b / AS-II c | fh | Ramírez, 2010 |  |
| Stenochilidae | 13 | *Colopea pusilla* Simon 1893 | sc | AS-II b / FS-II | fh | Griswold, 2007 |  |
|  |  | *Stenochilus hobsoni* Cambridge 1870 | sc | AS-II b / FS-II | fh | Forster & Platnick, 1984 |  |
| Stiphidiidae | 135 | *Cambridgea foliata* Koch 1872 | - | - / SB | wb | Griswold, 2006 |  |
|  |  | *Corasoides* sp. Butler 1929 | - | - / SB | wb | Griswold, 2007 |  |
|  |  | *Ischalea* sp. Koch 1872 | - | FS-II | fh | Griswold, 2005 |  |
|  |  | *Stiphidion facetum* Simon 1902 | - | FS-II | fh | Griswold, 2006 |  |
| Symphytognathidae | 66 | *Patu* sp. Marples 1951 | - | - | wb | Lopardo, 2006 |  |
| Synotaxidae | 82 | *Calcarsynotaxus longipes* Wunderlich 1995 | - | - / SB | wb | Griswold, 2007 |  |
|  |  | *Synotaxus* sp. Simon 1895 | - | - / SB | wb | Griswold, 2007 |  |
| Telemidae | 61 | *Usofila* sp. Keyserling 1895 | - | FS-II | wb | Griswold, 2006 |  |
| Tengellidae | 57 | *Anachemmis linsdalei* Platnick & Ubick 2005 | sc + ct | AS-II b | fh | Platnick & Ubick, 2005 |  |
|  |  | *Liocranoides unicolor* Keyserling 1881 | sc + ct | AS-I a / AS-II b | fh | Ramírez, 2010 |  |
|  |  | *Tengella radiata* Kulczynski 1909 | sc | AS-I a / FS-II | wb | Ramírez, 2010 |  |
| Tetrablemmidae | 142 | *Perania nasuta* Schwendinger 1989 | - | SB | fh (?) | Ramírez, 2010 |  |
| Tetragnathidae | 957 | *Glenognatha foxi* McCook 1894 | - | - / SB | wb | Benjamin & Hormiga, 2010 |  |
|  |  | *Leucauge venusta* Walckenaer 1842 | - | - / SB | wb | Benjamin & Hormiga, 2010 |  |
|  |  | *Tetragnatha versicolor* Walckenaer 1842 | - | - / SB | wb | Benjamin & Hormiga, 2010 |  |
| Theraphosidae | 939 | *Aphonopelma seemanni* Cambridge 1897 | sc + ct | AS-II b | fh | Niederegger & Gorb, 2006 | Kottsieper, 2011 |
|  |  | *Avicularia* sp. Lamarck 1818 | sc + ct | AS-II b | fh | this study |  |
|  |  | *Brachypelma auratum* Schmidt 1992 | sc + ct | n. a. | fh | Rind et al., 2011 |  |
|  |  | *B. emilia* White 1856 | sc + ct | AS-II b | fh | Kottsieper, 2011 |  |
|  |  | *B. vagans* Ausserer 1875 | sc + ct | AS-II b | fh | this study |  |
|  |  | *Chromatopelma cyanopubescens* Strand 1907 | sc + ct | AS-II b | fh | Kottsieper, 2011 |  |
|  |  | *Grammostola aureostriata* Schmidt & Bullmer 2001 | sc + ct | AS-II b | fh | this study | Dunlop, 1995 |
|  |  | *Grammostola rosea* Walckenaer 1837 | sc + ct | AS-II b | fh | Rind et al., 2011 | Dunlop, 1995 |
|  |  | *Nhandu chromatus* Schmidt 2004 | sc + ct | AS-II b | fh | Kottsieper, 2011 |  |
|  |  | *Phormictopus cubensis* Chamberlin 1917 | sc + ct | AS-II b | fh | this study |  |
|  |  | *Poecilotheria regalis* Pocock 1899 | sc + ct | AS-II b | fh | this study |  |
|  |  | *Sericopelma rubronitens* Ausserer 1875 | sc + ct | AS-II b | fh | this study |  |
| Theridiidae | 2350 |  | - | n. a. / SB | wb | no data | Joqué & Dippenaar-Schoeman, 2007 |
| Theridiosomatidae | 89 |  | - | n. a. / SB | wb | no data | Joqué & Dippenaar-Schoeman, 2007 |
| Thomisidae | 2152 | *Aphantochilus rogersi* Cambridge 1870 | ct | FS-II / AS-II b | fh | this study | Ramírez, 2010 |
|  |  | *Borboropactus* sp. Simon 1884 | f-ct | FS-II / AS-I a | fh | Ramírez, 2010 |  |
|  |  | *Bucranium taurifrons* Cambridge 1887 | ct | FS-II / AS-II b | fh | this study |  |
|  |  | *Cupa kalawitana* Barrion & Litsinger 1995 | sc + f-ct | AS-I a | fh | Ramírez, 2010 |  |
|  |  | *Diaea dorsata* Fabricius 1777 | f-ct | - / AS-I a | fh | this study |  |
|  |  | *Ebrechtella tricuspidata* Fabricius 1775 | - | - / FS-II | fh | Wolff & Gorb, 2012c |  |
|  |  | *Epicadinus trifidus* Cambridge 1893 | f-ct | - / AS-I a | fh | this study |  |
|  |  | *Misumena vatia* Clerck 1757 | - | - / FS-II | fh | this study |  |
|  |  | *Onocolus pentagonus* Keyserling 1880 | f-ct | - / AS-I a | fh | this study |  |
|  |  | *Ozyptila praticola* Koch 1837 | f-ct | - / AS-I a | fh | this study |  |
|  |  | *Pistius truncatus* Pallas 1772 | f-ct | - / AS-I a | fh | this study |  |
|  |  | *Stephanopis ditissima* Nicolet 1849 | sc + f-ct | AS-I a | fh | Ramírez, 2010 |  |
|  |  | *Stephanopoides sexmaculata* Mello-Leitao 1929 | sc + f-ct | AS-I a | fh | Ramírez, 2010 |  |
|  |  | *Strophius albofasciatus*  Mello-Leitao 1929 | f-ct | - / AS-I a | fh | Ramírez, 2010 |  |
|  |  | *S. hirsutus* Cambridge 1891 | f-ct | - / AS-I | fh | this study |  |
|  |  | *Thomisus onustus* Walckenaer 1805 | - | - / FS-II | fh | Ramírez, 2010 |  |
|  |  | *Tmarus holmbergi* Schiapelli & Gerschman 1941 | f-ct | - / AS-I a | fh | Ramírez, 2010 |  |
|  |  | *T. studiosus* Cambridge 1892 | f-ct | - / AS-I a | fh | this study |  |
|  |  | *Xysticus cristatus* Clerck 1757 | f-ct | FS-II / AS-I a | fh | this study | Ramírez, 2010 |
|  |  | *X. lanio* Koch 1835 | f-ct | FS-II / AS-I a | fh | this study |  |
| Titanoecidae | 53 | *Goeldia* sp. Keyserling 1891 | - | FS-II | wb | Griswold, 2010 |  |
| Trechaleidae | 118 | *Trechalea* sp. Thorell 1869 | - | FS-II | fh | this study |  |
|  |  | *T. longitarsis* Koch 1847 | - | - | fh | Sierwald, 2008 |  |
| Trochanteriidae | 152 | *Doliomalus cimicoides* Nicolet 1849 | sc | AS-II b / - | fh | Ramírez, 2006 |  |
|  |  | *Fissarena ethabuka* Henschel et al. 1995 | sc + ct | AS-II b | fh | Henschel et al., 1995 |  |
|  |  | *Platyoides walteri* Karsch 1886 | sc | AS-II b / - | fh | Ramírez, 2010 |  |
|  |  | *Trachycosmus sculptilis* Simon 1893 | sc + ct | AS-II b | fh | Ramírez, 2010 |  |
| Uloboridae | 266 | *Uloborus glomosus* Walckenaer 1842 | - | FS-II / SB | wb | Ramírez, 2002 |  |
| Zodariidae | 970 | *Cryptothele alluaudi* Simon 1893 | - | FS-II | fh | Ramírez, 2010 |  |
|  |  | *Cybaeodamus enigmaticus* Mello-Leitao 1939 | - | FS-II | fh | Ramírez, 2010 |  |
|  |  | *Cyrioctea aschaensis* Schiapelli & Gerschman 1942 | - | FS-II | fh | Ramírez, 2010 |  |
|  |  | *Platnickia elegans* Nicolet 1849 | - | FS-I | fh | Ramírez, 2010 |  |
|  |  | *Zodarion* sp. Walckenaer 1826 | - | FS-II | fh | this study |  |
| Zoridae | 79 | *Odo bruchi* Mello-Leitao 1938 | sc + f-ct | AS-II b | fh | Ramírez, 2010 |  |
|  |  | *Xenoctenus* sp. Mello-Leitao 1938 | sc | AS-II b / FS-II | fh | Ramírez, 2010 |  |
|  |  | *Zora spinimana* Sundevall 1833 | sc + ct | AS-I b | fh | Wolff & Gorb, 2012c | Ramírez, 2010 |
| Zorocratidae | 42 | *Zorocrates gnaphosoides* Cambridge 1892 | sc | AS-I a / FS-II | fh | Ramírez, 2010 |  |
| Zoropsidae | 86 | *Griswoldia acaenata* Griswold 1991 | sc + f-ct | AS-I a | fh | Griswold, 2006 |  |
|  |  | *Kilyana hendersoni* Raven & Stumkat 2005 | sc + ct | AS-I a / AS-II b | fh | Griswold, 2006 |  |
|  |  | *Phanotea sathegyna* Griswold 1994 | sc + f-ct | AS-I a | fh | Griswold, 2006 |  |
|  |  | *Zoropsis spinimana* Dufour 1820 | sc + ct | AS-I a | fh | Griswold, 2006 |  |

^1^ Number of described species belonging to the family, data by Platnick (2012).

^2^ Definition provided in the main document.

^3^ Classification characterized in S2; following Wolff and Gorb, 2012b. First dominant setal type in ventral tarsus, second dominant setal type in pretarsus (or distal margin of tarsus), if different. ‘-‘ means no specialized (structured) setae, ‘-e’ enlarged seta (width of the distal lamella > 20 µm).

**^4^** fh, vagrant, free ambushing or sensing web builder; wb, prey capture web builder or silk thrower (see definitions in the Methods section of the main manuscript); data obtained from Joqué and Dippenaar-Schoeman (2007) and Cardoso et al. (2011), if no further references are declared

**References**

**Baehr, B. C. and Smith, H. M.** (2008). Three new species of the Australian orsolobid spider genus *Hickmanolobus* (Araneae: Orsolobidae). *Records of the Western Australian Museum* **24**, 325-336.

**Baehr, B. C. and Ubick, D.** (2010). A Review of the Asian Goblin Spider Genus *Camptoscaphiella* (Araneae: Oonopidae). **3697**, 1-65.

**Benjamin, S. P. and Hormiga, G.** (2010). Contribution to morphbank :: biological imaging. http://www.morphbank.net

**Bond, J.** (2010). Contribution to morphbank :: biological imaging. http://www.morphbank.net

**Bosselaers, J. and Joqué, R.** (2000). *Hortipes*, a huge genus of tiny afrotropical spiders (Araneae, Liocranidae). *Bull. AMNH* **256**, 1-108.

**Brescovit, A. D., Bertoncello, L. A., Ott, R. and Lise A. A.** (2004). Description and ecology of two new species of the Brazilian spider genus *Losdolobus* Platnick & Brescovit (Araneae, Dysderoidea, Orsolobidae). *Revista Ibérica de Aracnología* **9**, 249-257.

**Cardoso, P., Pekár, S., Jocqué, R., Coddington, J. A.** (2011) Global Patterns of Guild Composition and Functional Diversity of Spiders. PLoS One 6: e21710. doi:10.1371/journal.pone.0021710

**Coddington, J. A.** (2010). Contribution to morphbank :: biological imaging. http://www.morphbank.net

**Davila, D. S.** (2003). Higher-level relationships of the spider family Ctenidae (Araneae: Ctenoidea). *Bull. AMNH* **274**, 1-86.

**Dias, S. C. and Brescovit, A. D.** (2004). Microhabitat selection and co-occurrence of *Pachistopelma rufonigrum* Pocock (Araneae, Theraphosidae) and *Nothroctenus fuxico* sp. nov. (Araneae, Ctenidae) in tank bromeliads from Serra de Itabaiana, Sergipe, Brazil. *Rev. Bras. Zool.* **21**, 789-796.

**Dunlop, J. A.** (1995). Movements of scopulate cts at the tip of a tarantula spider. *Netherlands J. Zool.* **45**, 513-520.

**Fannes, W.** (2010). On Melchisedec, a new genus of the family Oonopidae (Araneae, Dysderoidea). *American Museum Novitates* **3702**, 1-28.

**Foelix, R. F.** (1970). Structure and function of tarsal sensilla in the spider Araneus diadematus*. J. Exp. Zool.* **175**, 99-124.

**Foelix, R. F. and Chu-Wang, I.-W.** (1975). The structure of scopula hairs in spiders. *Proc. 6^th^ Int. Arachnol. Congr.* 1974, Free University Amsterdam, pp. 156-157.

**Foelix, R. F., Jackson, R. R., Henksmeyer, A. and Hallas, S.** (1984). Tarsal hairs specialized for prey capture in the salticid *Portia*. *Rev. Arachnol.* **5**, 329-334.

**Foelix, R. F., Erb, B. and Michalik, P.** (2010). Scopulate hairs in male *Liphistius* spiders: probable contact chemoreceptors. *J. Arachn.* **38**, 599-603.

**Foelix, R. F. and Erb, B.** (2011). Microscopical studies on exuviae of the jumping spider *Phidippus regius.* *PECKHAMIA* **90**, 1-15.

**Forster, R. R.** (1970). The spiders of New Zealand. Part III: Desidae, Dictynidae, Hahniidae, Amaurobioididae, Nicodamidae. Otago Museum Trust board, 187 pp.

**Forster, R. R. and Platnick, N.** (1984). A review of the archaeid spiders and their relatives, with notes on the limits of the superfamily Palpimanoidea (Arachnida, Araneae). Bull. AMNH, 178, 1-106.

**Fourie, R, Haddad, C. R. and Joqué, R.** (2011). A revision of the purse-web spider genus *Calommata* Lucas, 1837 (Araneae, Atypidae) in the Afrotropical Region. *ZooKeys* **95**, 1-28.

**Grismado, C. J.** (2010). Description of *Birabenella,* a New Genus of Goblin Spiders from Argentina and Chile (Araneae: Oonopidae). *American Museum Novitates* **3693**, 1-21.

**Griswold, C. and Platnick, N. I.** (1987). On the First African Spiders of the Family Orsolobidae (Araneae, Dysderoidea). *American Museum Novitates* **2892**, 1-14.

**Griswold, C.** (2005). Contribution to morphbank :: biological imaging. http://www.morphbank.net

**Griswold, C.** (2006). Contribution to morphbank :: biological imaging. http://www.morphbank.net

**Griswold, C.** (2007). Contribution to morphbank :: biological imaging. http://www.morphbank.net

**Griswold, C.** (2008). Contribution to morphbank :: biological imaging. http://www.morphbank.net

**Griswold, C.** (2010). Contribution to morphbank :: biological imaging. http://www.morphbank.net

**Haddad, C. R., Lyle, R., Bosselaers, J. and Ramírez, M.** (2009). A revision of the endemic South African spider genus *Austrachelas*, with its transfer to the Gallieniellidae (Arachnida: Araneae). *Zootaxa* **2296**, 1–38.

**Hill, D. E.** (1977). The pretarsus of salticid spiders. *Zool. J. Linn. Soc.* **60**, 319-338.

**Izquierdo, M. A. and Labarque**, F. M. (2010). Description of the female of *Orsolobus pucara* Forster & Platnick 1985, with comments on the functional morphology of the female genitalia in Dysderoidea. *J. Arachn.* **38**,511-520.

**Joqué, R. and Dippenaar-Schoeman, A. S.** (2007). *Spider families of the world.* 2^nd^ Edition. Royal Museum for Central Africa, Tervuren.

**Kesel, A. B., Martin, A. and Seidl, T.** (2003). Adhesion measurements on the attachment devices of the jumping spider *Evarcha arcuata*. *J. Exp. Biol.* **206**, 2733-2738

**Knoflach, B., Pfaller, K. and Stauder, F.** (2009). *Cortestina thaleri* – a new dwarf six-eyed spider from Austria and Italy (Araneae: Oonopidae: Oonopinae). *Contrib. Nat. Hist.* **12**, 743–771.

**Kottsieper, J.** (2011). Functional morphology and biomechanics of the attachment system in mygalomorph spiders. Diploma Thesis, University of Kiel, Zoological Institute.

**Lapinski, W.** (2009). Habitat use and coexistence in an assemblage of Neotropical wandering spiders. Diploma Thesis, University of Ulm, Institute for Experimental Ecology (Biologie III).

**Logunov, D. V.** (1996). Salticidae of Middle Asia. 3. A new genus, *Proszynskiana* gen. n., in the subfamily Aelurillinae (Araneae, Salticidae). *Bull. Br. arachnol. Soc.* **10**, 171–177.

**Lopardo, L.** (2006). Contribution to morphbank :: biological imaging. http://www.morphbank.net

**Miller, G. L., Miller, P. R. and Brady, A. R.** (1988). Adhesive hairs in lycosid spiders of various life styles, including the occurrence of cts in *Lycosa hentzi* Banks. *Bull. Br. arachnol. Soc.* **7**, 213-216.

**Moon, M. J. and Park, J. G.** (2009). Fine structural analysis on the dry adhesion system of the jumping spider *Plexippus setipes* (Araneae: Salticidae). *Animal Cells Syst.* (Seoul) **13**, 161-167.

**Muster, C.** (2009). The *Ebo*-like running crab spiders in the Old World (Araneae, Philodromidae). *ZooKeys* **16**, 47-73.

**Niederegger, S. and Gorb, S. N.** (2006). Friction and adhesion in the tarsal and metatarsal scopulae of spiders. *J. Comp. Physiol. A* **192**, 1223-1232.

**Platnick, N. I. and Penney, D.** (2004). A Revision of the Widespread Spider Genus *Zimiris* (Araneae, Prodidomidae). *American Museum Novitates* **3450**, 1-12.

**Platnick, N. I., Shadab, M. U. and Sorkin, L. N.** (2005). *American Museum Novitates* **3499**, 1-31.

**Platnick, N. I. and Ubick, D.** (2005). A Revision of the North American Spider Genus *Anachemmis* Chamberlin (Araneae, Tengellidae). *American Museum Novitates* **3477**, 1-20.

**Platnick, N. I. and Dupérré, N.** (2009). The Goblin Spider Genus *Heteroonops* (Araneae, Oonopidae), With Notes on *Oonops*. *American Museum Novitates* **3672**, 1-72.

**Platnick, N. I.** (2012). The world spider catalog. Version 13.0. American Museum of Natural History, New York. http://research.amnh.org/iz/spiders/catalog/COUNTS.html

**Rambla, M.** (1990). Les scopula des Opilions, differences avec les scopula des Araignées (Arachnida, Opiliones, Araneae). *Bull. Soc. Europ. Arachnol.* **1**, 293-298.

**Ramírez, M. J.** (1995). A phylogenetic analysis of the subfamilies of Anyphaenidae (Arachnida, Araneae). *Ent. Scand.* **26**, 361-384.

**Ramírez, M. J.** (2002). Contribution to morphbank :: biological imaging. http://www.morphbank.net

**Ramírez, M. J.** (2004). Contribution to morphbank :: biological imaging. http://www.morphbank.net

**Ramírez, M. J.** (2006). Contribution to morphbank :: biological imaging. http://www.morphbank.net

**Ramírez, M. J.** (2007). Contribution to morphbank :: biological imaging. http://www.morphbank.net

**Ramírez, M. J.** (2009). Contribution to morphbank :: biological imaging. http://www.morphbank.net

**Ramírez, M. J.** (2010). Contribution to morphbank :: biological imaging. http://www.morphbank.net

**Reyes, A. X. G., Corronca, J. A. and Cava, M. B.** (2010). New species of *Unicorn* Platnick & Brescovit (Araneae, Oonopidae) from North-West Argentina. *Mun. Ent. Zool.* **5**, 374-379.

**Rind, F. C., Birkett, C. L., Duncan, B.-J. A. and Ranken, A. J.** (2011). Tarantulas cling to smooth vertical surfaces by secreting silk from their feet. *J. Exp. Biol.* **214**, 1874-1879.

**Rovner, J. S.** (1978). Adhesive hairs in spiders: behavioral functions and hydraulically mediated movement. *Symp. zool. Soc. Lond.* **42**, 99-108.

**Scharff, N.** (2007). Contribution to morphbank :: biological imaging. http://www.morphbank.net

**Scharff, N.** (2010). Contribution to morphbank :: biological imaging. http://www.morphbank.net

**Sierwald, P.** (2008). Contribution to morphbank :: biological imaging. http://www.morphbank.net

**Ubick, D. and Vetter, R. S.** (2005). A new species of *Apostenus* from California, with notes on the genus (Araneae, Liocranidae). *J. Arachn.* **33**, 63-75.

**Ubick, D. and Griswold, C. E.** (2011). The Malagasy goblin spiders of the new genus *Malagiella* (Araneae, Oonopidae). *Bull. AMNH* **356**, 1-86.

**Wanless, F. R.** (1978). Notes on spiders of the family Salticidae. 1. The genera *Spartaeus*, *Mintonia* and *Taraxella*. *Bull. Br. Mus. nat. Hist. (Zool.)* **52**, 107-137.

**Warui, C. and Joqué, R.** (2002). The first Gallieniellidae (Araneae) from Eastern Africa. *J. Arachn.* **30**, 307-315.

**Wolff, J. O. and Gorb, S. N.** (2012b). Surface roughness effects on attachment ability of the spider *Philodromus dispar* (Araneae, Philodromidae). *J. Exp. Biol.* **215**, 179-184.

**Wolff, J. O. and Gorb, S. N.** (2012c). Comparative morphology of pretarsal scopulae in eleven spider families. *Arthr. Struct. Devel.* **41**, 419-433.
