# Supplementary material for: The Great Silk Alternative: Multiple Co-Evolution of Web Loss and Sticky Hairs in Spiders
Source: PLoS One. 2013 May 1;8(5):e62682. doi: 10.1371/journal.pone.0062682 (PMC3641104; doi:10.1371/journal.pone.0062682)
Supplement: Figure S2 — Evolution of setal types in the Araneomorphae. A. Tree combining character traces from Ancestral State Analysis on dominant setal type of tarsus or pretarsus, if (false) claw tuft present. Blue shades indicate the highly anisotropic structured, but spatula lacking ‘frictional’ setae (FS). Yellow and red shades mark the adhesive setae of different types (AS). Purple lines indicate the serrated bristles (SB), being setal adaptations of the derived web building taxa. Setal type abbreviations are listed in detail in Fig. S1. B. Hypothesized evolutionary steps on setal morphology. The upper lineages assume independent evolution of adhesive pads in the pretarsus, whereas, in the lower ones, scopulae evolved first, then extended to the pretarsus, followed by claw tuft formation. Assumptions are based on the morphological comparison of scopula and claw tuft setae. (PDF) [file pone.0062682.s002.pdf]

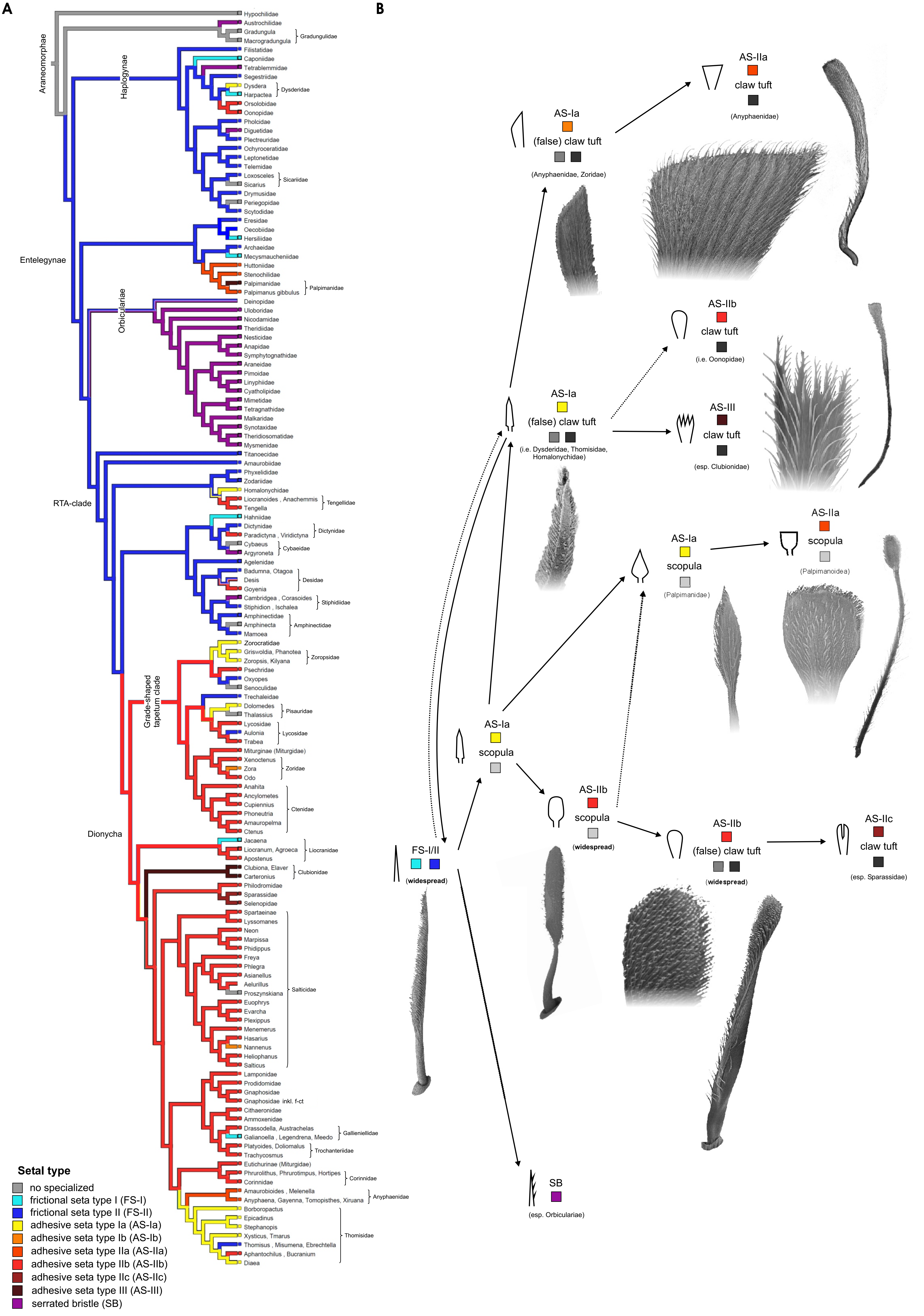

A
B
Araneomorphae
Haplogynae
Entelegynae
Orbiculariae
RTA-clade
Grade-shaped tapetum clade
Dionycha
Hypochilidae
Austrochilidae
Gradungula
Macrogradungula
Gradungulidae
Filistatidae
Caponiidae
Tetrablemmidae
Segestriidae
Dysdera
Harpactea
Dysderidae
Orsolobidae
Oonopidae
Pholcidae
Diguetidae
Plectreuridae
Ochyroceratidae
Leptonetidae
Telemidae
Loxosceles
Sicarius
Sicariidae
Drymusidae
Periegopidae
Scytodidae
Eresidae
Oecobiidae
Hersiliidae
Archaeidae
Mecysmaucheniidae
Huttoniidae
Stenochilidae
Palpimanidae
Palpimanus gibbulus
Palpimanidae
Deinopidae
Uloboridae
Nicodamidae
Theridiidae
Nesticidae
Anapidae
Symphytognathidae
Araneidae
Pimoidae
Linyphiidae
Cyatholipidae
Mimetidae
Tetragnathidae
Malkaridae
Synotaxidae
Theridiosomatidae
Mysmenidae
Titanoecidae
Amaurobiidae
Phyxelididae
Zodariidae
Homalonychidae
Liocranoides , Anachemmis
Tengella
Tengellidae
Hahniidae
Dictynidae
Paradictyna , Viridictyna
Dictynidae
Cybaeus
Argyroneta
Cybaeidae
Agelenidae
Badumna, Otagoa
Desis
Goyenia
Desidae
Cambridgea , Corasoides
Stiphidion , Ischalea
Stiphidiidae
Amphinectidae
Amphinecta
Mamoea
Amphinectidae
Zorocratidae
Griswoldia, Phanotea
Zoropsis, Kilyana
Zoropsidae
Psechridae
Oxyopes
Senoculidae
Trechaleidae
Dolomedes
Thalassius
Pisauridae
Lycosidae
Aulonia
Trabea
Lycosidae
Miturginae (Miturgidae)
Xenoctenus
Zora
Odo
Zoridae
Anahita
Ancylometes
Cupiennius
Phoneutria
Amauropelma
Ctenus
Ctenidae
Jacaena
Liocranum, Agroeca
Apostenus
Liocranidae
Clubiona, Elaver
Carteronius
Clubionidae
Philodromidae
Sparassidae
Selenopidae
Spartaeinae
Lyssomanes
Neon
Marpissa
Phidippus
Freya
Phlegra
Asianellus
Aelurillus
Proszynskiana
Euophrys
Evarcha
Plexippus
Menemerus
Hasarius
Nannenus
Heliophanus
Salticus
Salticidae
Lamponidae
Prodidomidae
Gnaphosidae
Gnaphosidae inkl. f-ct
Cithaeronidae
Ammoxenidae
Drassodella, Austrachelas
Galianoella , Legendrena, Meedo
Gallieniellidae
Platyoides, Doliomalus
Trachycosmus
Trochanteriidae
Eutichurinae (Miturgidae)
Phrurolithus, Phrurotimpus, Hortipes
Corinnidae
Corinnidae
Amaurobioides , Melenella
Anyphaena, Gayenna, Tomopisthes, Xiruana
Anyphaenidae
Borboropactus
Epicadinus
Stephanopis
Xysticus, Tmarus
Thomisus , Misumena, Ebrechtella
Aphantochilus , Bucranium
Diaea
Thomisidae
Setal type
no specialized
frictional seta type I (FS-I)
frictional seta type II (FS-II)
adhesive seta type Ia (AS-Ia)
adhesive seta type Ib (AS-Ib)
adhesive seta type IIa (AS-IIa)
adhesive seta type IIb (AS-IIb)
adhesive seta type IIc (AS-IIc)
adhesive seta type III (AS-III)
serrated bristle (SB)
AS-Ia
(false) claw tuft
(Anyphaenidae, Zoridae)
AS-IIa
claw tuft
(Anyphaenidae)
AS-IIb
claw tuft
(i.e. Oonopidae)
AS-Ia
(false) claw tuft
(i.e. Dysderidae, Thomisidae, Homalonychidae)
AS-III
claw tuft
(esp. Clubionidae)
AS-IIa
scopula
(Palpimanoidea)
AS-Ia
scopula
(Palpimanidae)
AS-Ia
scopula
AS-IIb
scopula
(widespread)
AS-IIb
(false) claw tuft
(widespread)
AS-IIc
claw tuft
(esp. Sparassidae)
FS-I/II
(widespread)
SB
(esp. Orbiculariae)
