# Supplementary material for: The Great Silk Alternative: Multiple Co-Evolution of Web Loss and Sticky Hairs in Spiders
Source: PLoS One. 2013 May 1;8(5):e62682. doi: 10.1371/journal.pone.0062682 (PMC3641104; doi:10.1371/journal.pone.0062682)
Supplement: Figure S3 — Character states recorded for convergently evolved adhesive setae in the Araneae. (DOC) [file pone.0062682.s003.doc]

S3. Ultrastructural character states in the convergently evolved adhesive setae among the Araneae.

|  | Mygalomorphae | Dysderoidea | Palpimanoidea | derived RTA-clade | Thomisidae | Desidae | Homalonychidae | Tengellidae |
| --- | --- | --- | --- | --- | --- | --- | --- | --- |
| *types* | Ia, IIb | Ia, IIb | Ia, IIa / b, III | all | Ia, IIb | IIb | Ia | IIb |
| *shape* | spatulate | brushlike / spatulate / lamelliform | spatulate / lamelliform | spatulate / lamelliform | lamelliform | lamelliform | brushlike | lamelliform |
| *backing microripples* | + | - / (+) | + | + | - | n.a. | - | n.a. |
| *backing microtrichia* | + | - | + | + | - | + | - | + |
| *spatula shape* | triangular / circular | triangular / circular | triangular / ellipsoid | triangular | triangular / reduced | triangular | triangular | triangular |
| *twisted shaft in claw tuft setae* | - | - / + | - | + / (-) | - | - | - / + | - |
| *cross section of distal part* | lamellate | cylindrical / lamellate | lamellate | lamellate | cylindrical | lamellate | cylindrical | lamellate |
| *tenent plate of claw tufts* | plate-like | plate-like / onychium | claw basis | plate-like | plate-like | claw basis | plate-like | plate-like |
